# Cryptoglandular Anal Fistula Core Outcome Measurement Set (AFCOMS): standardised definitions and measurement instruments

**DOI:** 10.1016/j.eclinm.2025.103745

**Published:** 2026-02-06

**Authors:** Nedim Tabakovic, Shivani Joshi, Merel Kimman, Litza Mitalas, Nusrat Iqbal, Phil Tozer, Stéphanie Breukink, AFCOMS Consensus Meeting Collaborators, Ademola Adeyeye, Andrea Marco Tamburini, Angelo Alessandro Marra, Anthony Lin, Arda Isik, Eleni Andriopoulou, Fatima Senra, Flavia Alexandre, Francesco Pata, Gaetano Gallo, Gabriele Bislenghi, Jasper Stijns, Jennie Grainger, Jesus Lopez-Alcalde, Johannes Jongen, Lilli Lundby, Lillian Reza, Martijn Gosselink, Peter Ambe, Philip Lung, Raimund Strouhal, Ricardo Rocha, Giulio Aniello Santoro, Stephen Ward

**Affiliations:** a Department of Surgery and Colorectal Surgery, Maastricht University Medical Centre+, the Netherlands; b Institute NUTRIM for Nutrition and Translational Research in Metabolism, Faculty of Health, Medicine & Life Sciences, Maastricht University, the Netherlands; c Robin Phillips' Fistula Research Unit, St Mark's Hospital, London, UK; d Department of Clinical Epidemiology and Medical Technology Assessment, Care and Public Health Research Institute (CAPHRI), Maastricht University Medical Centre+, the Netherlands; e GROW Research Institute for Oncology and Reproduction, Faculty of Health, Medicine & Life Sciences, Maastricht University, the Netherlands

**Keywords:** Cryptoglandular anal fistula, Core outcome set, Patient-reported outcomes, Proctology, Delphi consensus

## Abstract

**Background:**

Cryptoglandular anal fistula is a condition that significantly impairs quality of life in patients. Despite the recent development of the Anal Fistula Core Outcome Set (AFCOS), which identified ten key outcomes, variation in outcome definitions and measurement instruments hampers comparability across studies and limits evidence synthesis. An essential final step to improve future comparability is the development of a Core Outcome Measurement Set (COMS) aligned with AFCOS; this study aimed to establish such a COMS through an international, consensus-driven process.

**Methods:**

This study was conducted in three phases according to the Core Outcome Measures Effectiveness Trials (COMET) methodology. Phase 1 included a scoping review to identify definitions and measurement instruments for all AFCOS outcomes. Phase 2 involved summarising available evaluation of psychometric properties and overall feasibility of each instrument according to the COSMIN criteria. Phase 3 consisted of an international two-round Delphi survey conducted from September 2023 until May 2024 and a final consensus meeting in June 2024 with patients and healthcare professionals to agree on definitions, measurement instruments and timepoints.

**Findings:**

In Delphi round 1, 92 of 110 participants (85 health professionals, 7 patients, from 18 countries) completed the survey (84% overall response). Many instruments had insufficient content validity when evaluated by patients. In round 2, 70 of 76 participants (63 professionals, 7 patients) completed the survey (92% response). A final consensus meeting was attended by 27 participants (26 clinicians and 1 patient representative). Clinical fistula healing was defined as the absence of discharge symptoms, abscess, infection or inflammation, with no recurrence or persistence for ≥6 months. Recurrence was defined as the reappearance of symptoms after this healing period. Radiological healing was defined as complete resolution of any visible fistula tract and inflammatory mass, ± fibrosis on MRI. Development of an additional fistula was defined as a separate, anatomically distinct tract. Complications were classified according to the Clavien-Dindo system, and reinterventions were limited to surgical or radiological procedures. The Anal Fistula Quality of Life Scale was selected to assess quality of life, fistula symptoms and psychological impact, the Vaizey score to assess continence, and a numerical rating scale to assess patient satisfaction. Timepoints were set at 3- and 12-months post-treatment.

**Interpretation:**

AFCOMS provides standardised outcome definitions and measurement tools for use in future cryptoglandular anal fistula research, enhancing reporting consistency and enabling evidence synthesis.

**Funding:**

There was no external funding for this study. The study was conducted independently by the authors.

**Research in context**

**Evidence before this study**

A literature search was conducted to identify definitions and measurement instruments for outcomes included in the Anal Fistula Core Outcome Set (AFCOS). Searches of Embase, Medline, and the Cochrane Library (January 1st 2008–August 31st 2022) used terms related to cryptoglandular anal fistula. Eligible studies enrolled at least ten patients, evaluated medical or surgical interventions, and reported at least one AFCOS-related outcome. The search revealed major heterogeneity in outcome definitions and measurement, with limited validation of patient-reported tools. Although AFCOS defined what to measure, no consensus existed on how or when outcomes should be assessed.

**Added value of this study**

This study finalises the outcome standardisation process by developing the Anal Fistula Core Outcome Measurement Set (AFCOMS). It establishes consensus-based definitions, instruments, and timepoints to improve consistency and relevance in anal fistula research.

**Implications of all the available evidence**

Together, AFCOS and AFCOMS provide a complete framework for outcome reporting in anal fistula studies. Their adoption may reduce heterogeneity, facilitate evidence synthesis, and support development of future evidence-based guidelines. Future work should validate instruments across diverse populations, translate them for broader use, and assess feasibility in low-resource settings.

## Introduction

Cryptoglandular anal fistulas are abnormal, non-anatomical tracts between the anal canal and perianal skin, affecting approximately 1.2–2.8 per 10,000 individuals annually.^1^ Most commonly arising as sequelae of anorectal abscesses originating at the anal glands,^2^ these fistulas can have a devastating impact on patients’ daily functioning and emotional wellbeing. The combination of persistent discharge, pain, and incontinence, combined with the risk of recurrence and long-term morbidity, substantially impair quality of life of these patients.^3^ Treatment is notoriously difficult, with reported healing rates ranging from 60 to 70%, depending on fistula complexity and surgical technique.^4^

Despite a broad spectrum of surgical options, identifying the optimal treatment for individual patients remains challenging due to inconsistent reporting and poor methodological quality of published studies, which limits evidence-based decision making. This problem is reflected in the European guideline for cryptoglandular anal fistula, where only four out of 42 recommendations are supported by a moderate level of evidence, and the majority rely on expert opinion.^5^ A primary contributor to this evidence gap is the considerable heterogeneity in outcome definitions and measurement instruments used across studies.^6^

To address this challenge, and the problem of historically clinician-centred markers of success, Core Outcome Sets (COS) have been proposed as a standardised framework to ensure that all clinical trials for a specific condition report a minimum set of critical outcomes^7^ which all stakeholders have contributed to the selection of. In the context of anal fistula, the Anal Fistula Core Outcome Set (AFCOS) was recently developed through international consensus to prioritise outcomes most relevant to both patients and clinicians.^8^ While AFCOS provides clarity on what to measure, it does not define how and when these outcomes should be measured, an essential next phase to reduce variability and improve comparability in future research.

Core Outcome Measurement Sets (COMS), developed in accordance with COSMIN (COnsensus-based Standards for the selection of health Measurement INstruments) and COMET (Core Outcome Measures in Effectiveness Trials) guidelines,^9^^,^^10^ aim to establish consensus-based definitions and standardised measurement instruments for each core outcome of as set. This study builds on the established AFCOS by developing a COMS for cryptoglandular anal fistula, through a multi-stakeholder, international Delphi process and consensus meeting. The AFCOMS initiative is intended to improve consistency in outcome measurement, facilitate evidence synthesis, and support the development of evidence-based guidelines and patient-centred care pathways, not only for surgical intervention but also for non-surgical options, thereby enabling meaningful comparison across diverse treatment approaches.

## Methods

This study was conducted in line with established guidelines and personalised guidance from the COSMIN and COMET initiatives, and adhered to the DELPHISTAR reporting guidelines.

**Fig. 1:**
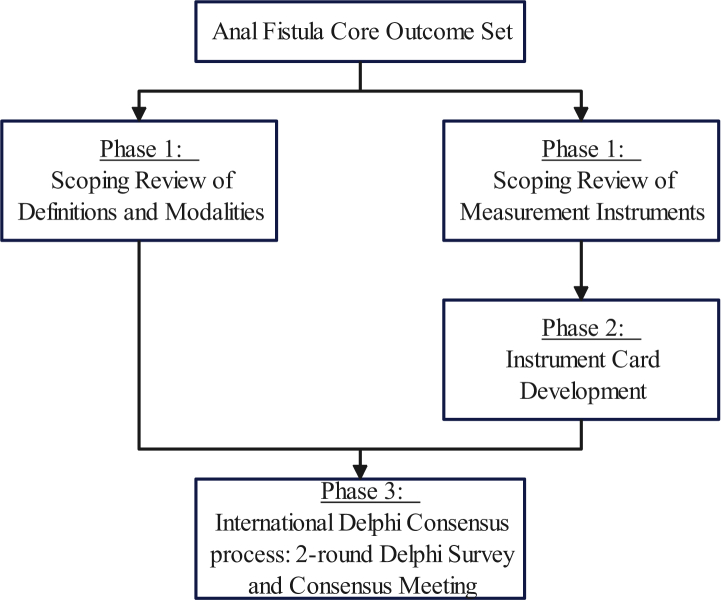
*Study phases and methodology overview*.

The study consisted of three phases, as outlined in Fig. 1:

### Phase 1: identification of definitions and measurement instruments

A scoping review was conducted to identify existing definitions and measurement instruments for outcomes included in the previously established Anal Fistula Core Outcome Set (AFCOS). The systematic review by Machielsen et al.,^6^ which covered studies published from January 2008 to May 2020, was updated to extend the search period to August 2022.

Studies were eligible if they included ≥10 adult patients with cryptoglandular anal fistula, evaluated medical or surgical interventions, and reported on at least one AFCOS-related outcome. Eligible study types included prospective and retrospective clinical studies, systematic reviews, and observational cohort studies. Two reviewers independently screened titles and abstracts, with discrepancies resolved through discussion. Data extraction focused on the outcomes assessed, outcome definitions, assessment modalities, measurement instruments used, and timing of assessments.

### Phase 2: creation of instrument cards

For each identified measurement instrument, we conducted a structured literature search in PubMed and Embase using a COSMIN-endorsed filter to appraise psychometric properties.^11^ This was supplemented by a grey literature search (e.g. instrument manuals, developer websites) to capture feasibility data.

We then developed standardised “instrument cards” to summarise key characteristics of each instrument in a user-friendly format.^12^ These cards included information on domains covered, scoring systems, mode of administration, training requirements, licencing constraints, equipment needs, and psychometric performance (validity, reliability, responsiveness) based on COSMIN criteria. Importantly, we also included the level of patient involvement in development. The cards served as decision-support tools during the Delphi process and were reviewed for clarity by patient partners.

### Phase 3: international Delphi consensus

A modified Delphi methodology was employed to achieve structured consensus among a diverse international panel of stakeholders, including both patients and healthcare professionals. This approach has several advantages including the capacity to incorporate global multidisciplinary opinion, reduce bias from dominant individuals, and facilitate anonymous voting.

#### Panellist recruitment

Healthcare professionals were recruited through targeted invitations to individuals involved in the original AFCOS development, members of the European Society of Coloproctology (ESCP) Perianal Fistula Guideline Committee, and members of the Association of Coloproctology of Great Britain and Ireland (ACPGBI) and the Dutch Society of Colorectal Surgery (DSCRS). An open call was also distributed via professional ESCP communication channels to engage the broader membership of ESCP. In order to widen the outreach of the survey, invited recipients were encouraged to share the invitation with colleagues who had relevant clinical or research expertise.

Patients were recruited via three methods: (1) participants from the previous AFCOS Delphi study who had consented to future contact, (2) a moderated English-language online peer support group for patients with anal fistula with around 1900 participants, and (3) direct invitations to 85 eligible patients attending the colorectal surgery outpatient clinic at Maastricht University Medical Centre. Patient-facing materials, including the Delphi survey and instrument cards, were reviewed for comprehensibility and translated into lay-friendly English and Dutch language by patient advocates within the study group.

#### Division of the AFCOS items and questionnaire set-up

**Fig. 2:**
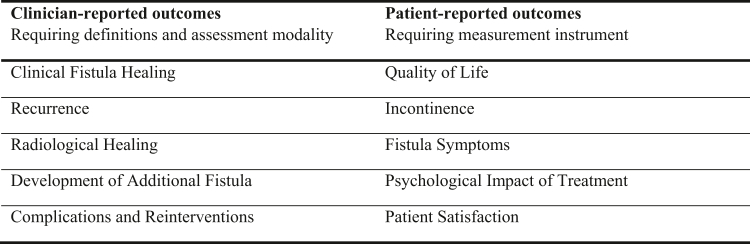
Overview of clinician-reported and patient-reported outcomes from the AFCOS.

Outcomes from the AFCOS were categorised as either clinician-reported or patient-reported. Clinician-reported outcomes required both a clear definition and a modality for assessment in clinical research or practice. Patient-reported outcomes required the selection of a measurement instrument. An overview of these outcomes is shown in Fig. 2.

Separate questionnaires were developed for healthcare professionals and patients. Healthcare professionals were asked to rate definitions and assessment modalities for clinician-reported outcomes, in addition to measurement instruments for patient-reported outcomes. Patients evaluated only the assessment modalities and measurement instruments. The patient questionnaire was made available in both Dutch and English and was specifically designed to be clear and comprehensible to lay audiences.

#### Online Delphi survey rounds 1 and 2

Two Delphi rounds were conducted online using the Qualtrics platform. Participants were asked to rate the proposed definitions, assessment modalities, and measurement instruments using the Grading of Recommendations Assessment, Development and Evaluation (GRADE) scale (1–9, with an additional “unable to score” option).^13^ The following consensus criteria were applied: (1) if ≥ 70% of both healthcare professionals and patients scored an item 7–9, it progressed to the next round; (2) if <70% of healthcare professionals scored an item 7–9 but the patient panel's median score was ≥7, it also progressed. Items with <70% agreement from healthcare professionals and a patient panel median <7 were excluded. This approach ensured that in cases where clinicians rated an instrument below the 70% threshold, strong patient endorsement (median ≥7) could overrule lower clinician input and secure its inclusion, and these thresholds were applied consistently in both rounds.^14^^,^^15^

During each round, participants were also given the opportunity to suggest additional definitions, assessment modalities, or measurement instruments they considered relevant that had not been identified in the preceding literature search, via free text comment fields provided for each outcome. Any newly suggested measurement instrument underwent rigorous evaluation and instrument card creation as per phase 2 to ensure they were consistently presented within the following round.

#### Consensus meeting

Participants who had completed both Delphi rounds were invited to the final international consensus meeting, held online via Microsoft Teams. Anonymous voting was facilitated via polleverywhere.com, and outcomes were presented in the same order as in the Delphi rounds to maintain consistency.

For clinician-reported outcomes, up to three variations of each definition were presented, based on submissions and ratings from the previous rounds. Voting was anonymous and results were only displayed to participants after voting had closed. Consensus was defined as ≥70% agreement for any given option. If this threshold was not reached, the two highest-rated options were shortlisted for a second, head-to-head vote. The same ≥70% threshold was applied in this second round. If consensus still was not achieved, the result was recorded as “no consensus.” Disagreements were addressed through facilitated discussion, led by a moderator who ensured balanced input and timekeeping. The patient participant was explicitly invited to provide input on each relevant outcome, and their perspective was given particular emphasis in areas with divergent views.

### Ethics

Ethical approval for this study was granted by the Maastricht University Medical Centre Ethical Committee (reference number: METC 2022-3466), and informed consent was obtained from all participants at the start of the online Delphi rounds.

### Statistics

Descriptive statistics were used to summarise the characteristics of participants in each Delphi round and the consensus meeting. Sex of participants was collected, but gender identity was not recorded. Because this study focused on achieving group consensus rather than analysing individual-level outcomes, sex-based differences were not explored.

For each item, the proportion of participants scoring 7–9 on the 1–9 GRADE scale was calculated separately for healthcare professionals and patients. “Unable to score” and missing responses were excluded from denominators for item-level percentages. Progression rules were prespecified: items advanced if at least 70% of both stakeholder groups scored 7–9, or if the patient median rating was 7 or higher when the clinician proportion was below 70%. Items with less than 70% clinician agreement and a patient median below 7 were excluded. At the consensus meeting, anonymous voting was conducted, and consensus was defined a priori as at least 70% agreement; if not achieved, the two highest-rated options entered a head-to-head vote using the same threshold. Analyses were performed descriptively using Microsoft Excel.

### Role of funding source

This study received no specific grant from any funding agency. The study was conducted independently by the authors.

## Results

### Phase 1: scoping review of definitions and measurement instruments

**Fig. 3:**
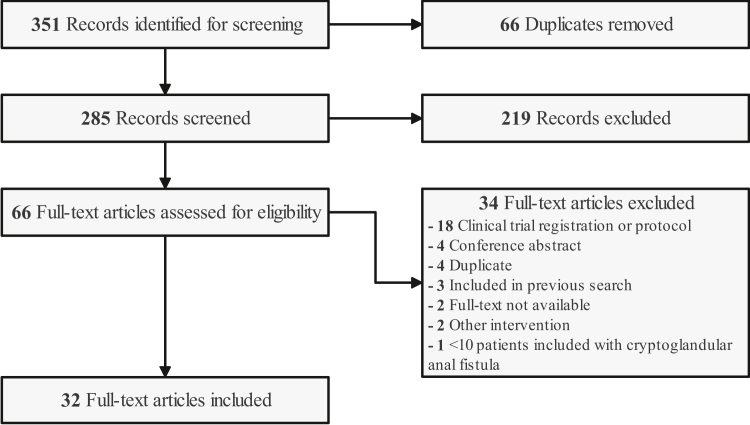
*PRISMA flow diagram: scoping review of definitions and measurement instruments*.

The updated search yielded 285 additional studies, of which 32 met the eligibility criteria and were included for data extraction. The process is summarised in Fig. 3.

For clinician-reported outcomes, no new definitions were identified for clinical or radiological healing. Updated data on the frequency and content of definitions and assessment modalities were extracted. Radiological healing was assessed in nine studies, but only five provided sufficient detail on its definition. Therefore, the proposed definitions for radiological healing were developed based on the existing Magnetic Resonance Imaging (MRI) activity-based scores for Crohn's anal fistula (e.g. Van Assche Index and MAGNIFI-CD) (see Supplementary S1 and S2).

For patient-reported outcomes, one additional measurement instrument, the Quality of Life Anal Fistula Questionnaire (QoLAF-Q), was identified for assessing quality of life. For all other patient-reported outcomes, updated frequency data were extracted from the newly included studies (see Supplementary S3).

### Phase 2: development of instrument cards

All instruments measuring patient-reported outcomes that were identified through the scoping review were evaluated for psychometric properties (if available) and feasibility in practice. Validation studies were identified through structured searches in PubMed and Embase using a COSMIN-endorsed filter. This was supplemented by a grey literature search, including developer websites and instrument manuals, to retrieve additional data on scoring, licencing, and administration requirements.

#### Search: validation studies

The search identified three relevant studies. One COSMIN-compliant evaluation study on the Quality of Life Anal Fistula Questionnaire (QoLAF-Q) provided data on validity, reliability, and responsiveness in a cryptoglandular fistula population. The other two studies described the development of the Gastrointestinal Quality of Life Index (GIQLI) and QoLAF-Q instruments. Importantly, both of these instruments were developed with direct input from patients with anal fistula. No validation studies were identified for the remaining instruments. Full search results are presented in the Appendix.

#### Instrument cards

**Fig. 4:**
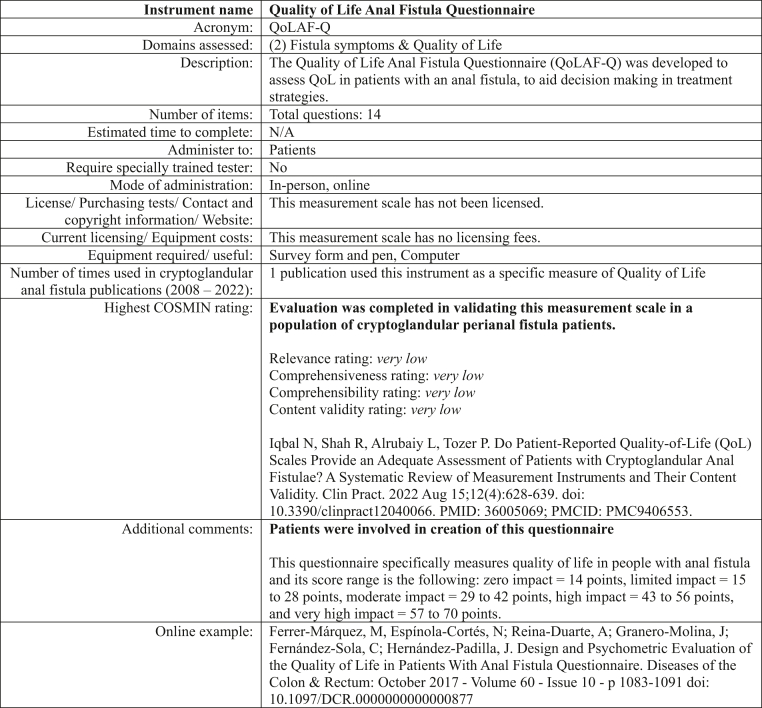
Instrument card for the Quality of Life Anal Fistula Questionnaire (QoLAF-Q).

To facilitate standardised comparison, we developed structured instrument cards summarising key features of each instrument. These included the domain(s) measured, number of items, administration method, scoring system, licencing constraints, equipment needs, and COSMIN-derived evidence on validity, reliability, and responsiveness. The cards also noted whether the instrument was developed with input from patients with cryptoglandular anal fistula. An example of a standardised instrument card (QoLAF-Q) is presented in Fig. 4. This illustrates the format and key data points reviewed by participants during the Delphi rounds. A summary table of all relevant instrument properties can be found in the Supplementary S10 and the full set of instrument cards is available in the Appendix.

### Phase 3: international Delphi consensus process

A two-round online Delphi survey was conducted to evaluate definitions, assessment modalities, and measurement instruments for each core outcome. Round 1 was held between September and October 2023, followed by Round 2 which took place between March and May 2024.

In Round 1, a total of 110 participants registered to participate, including 95 healthcare professionals (86%) and 15 patients (14%). Of these, 92 completed the questionnaire, resulting in an overall completion rate of 84%. Among healthcare professionals, 85 out of 95 completed the round, yielding a completion rate of 89%. Among patients, 7 out of 15 completed the questionnaire, corresponding to a completion rate of 47%. Free text comment fields within the survey identified patient difficulties in evaluating the measurement properties of instruments.

In Round 2, 76 participants took part, consisting of 69 healthcare professionals (91%) and 7 patients (9%). A total of 70 participants completed the questionnaire, resulting in an overall completion rate of 92%. This included a 100% completion rate among patients (7 of 7) and a 91% completion rate among healthcare professionals (63 of 69).

Participant demographics for both rounds are summarised in Supplementary S4 and countries of residence are shown in Supplementary S5.

The final consensus meeting was held on 17 June 2024 to determine the final Anal Fistula Core Outcome Measurement Set (AFCOMS). Twenty-seven participants attended, including 26 healthcare professionals (24 surgeons, one radiologist, and one clinical researcher) and one patient representative. Voting was conducted anonymously, and the consensus threshold was pre-defined at 70% agreement. Specific measures were taken to ensure that the patient perspective was meaningfully represented and considered during all stages of the meeting. Participant demographics for the consensus meeting can be seen in Table 1. We have not displayed extra demographic data for the sole patient representative that attended the meeting in order to maintain their anonymity.

**Table 1:** Demographics Consensus Meeting Healthcare professionals.

	Consensus meeting participants N = 26
**Sex**	
Male	20 (77)
Female	6 (23)
**Country**	
Argentina	1 (4)
Aruba	1 (4)
Austria	1 (4)
Belgium	2 (8)
Denmark	1 (4)
Germany	2 (8)
Italy	5 (19)
Netherlands	1 (4)
New Zealand	1 (4)
Nigeria	1 (4)
Portugal	1 (4)
Switzerland	1 (4)
Turkey	1 (4)
United Kingdom	7 (27)
**Years of practice/Experience**	
0–5	4 (15)
6–10	4 (15)
11–20	11 (42)
>20	7 (27)
**Volume of patients treated/seen annually**	
0–20	3 (12)
21–50	11 (42)
51–100	10 (38)
>100	2 (8)
**Work setting**	
General hospital/teaching hospital	9 (35)
Tertiary referral centre	15 (58)
Private Hospital	2 (8)

#### Delphi survey rounds: clinician-reported outcomes

For clinical fistula healing, three descriptive components met the predefined thresholds for progression to the consensus meeting: absence of discharge symptoms (84%), absence of abscess, infection, or inflammation (76%), and absence of persistence or recurrence (72%). For radiological healing, two components progressed: absence of an inflammatory mass (80%) and presence of fibrosis (73%). The outcome recurrence was conceptually defined as the return of symptoms following a documented period of clinical healing and was therefore not rated independently, as its definition was derived from that of clinical healing. For the exact scoring in both survey rounds, see Supplementary S6.

Regarding assessment modalities, clinical examination met the criteria for progression to the final consensus meeting as the preferred method for assessing clinical healing, with agreement from 95% of healthcare professionals and 85% of patients. MRI progressed as the preferred assessment modality for assessing radiological healing, supported by 95% of professionals and 77% of patients. Both MRI and clinical examination progressed to the consensus meeting as appropriate modalities for assessing recurrence and the development of additional fistulas, with agreement levels exceeding 80% across stakeholder groups. For the assessment of complications and reinterventions, clinical examination, history taking, and MRI all met the thresholds for progression, although agreement on MRI was somewhat lower among patients. During the Delphi survey rounds, participants also proposed via the comment fields that complications should be classified using a standardised system, and that the term “reintervention” should be limited to surgical and radiological procedures.

The scores of both survey rounds are demonstrated in Supplementary S7.

#### Delphi survey rounds: patient-reported outcomes

Four instruments met the predefined thresholds for progression to the consensus meeting for assessing quality of life: the FISI, FIQL, QoLAF-Q, and AF-QOL. The AF-QOL, which was newly proposed by participants during Round 1, was subsequently evaluated and developed into a full instrument card for inclusion in Round 2. For incontinence, the Vaizey, Wexner, COREFO, and FIQL met the criteria for progression to the final consensus meeting, with the Vaizey and Wexner scores receiving the highest ratings from patient participants. The Perianal Disease Activity Index (PDAI) progressed for evaluating fistula symptoms, and the Hospital Anxiety and Depression Scale (HADS) progressed for assessing psychological impact of treatment. Although originally suggested for the outcome quality of life, the AF-QOL's domains also covered fistula symptoms and the psychological impact of treatment. As a result, the AF-QOL was included in Delphi voting for all three outcomes and met the progression threshold in each case. The QoLAF-Q, which was identified during the literature review, was also suggested via the comment fields to be included in the voting options for symptoms and psychological impact of treatment, as with AF-QOL. Consequently, it reached the progression threshold for both fistula symptoms and psychological impact. No instrument met the threshold for progression for the outcome patient satisfaction.

An analysis was performed to examine the impact of weighting patient input in the online survey rounds. This showed that while instruments such as FISI, QoLAF-Q, AF-QOL, Wexner and Vaizey would have been included in the consensus meeting based on clinician votes alone, several other instruments were retained primarily due to strong patient endorsement despite weaker clinician support. This included FIQL in the quality-of-life domain, FIQL and COREFO in incontinence, PDAI in symptoms, and AF-QOL and HADS in psychological impact. The eventually chosen instruments were consistently supported throughout the online round and the AF-QOL would in the end have not been able to be selected for the domain psychological impact due to exclusion based on the clinician vote. This demonstrates that patient perspectives directly shaped the final instrument set for the consensus meeting. The full scores of both survey rounds and the results of the analysis are presented in Supplementary S8 and S9.

#### Consensus meeting: clinician-reported outcomes

##### Clinical fistula healing

During the consensus meeting, agreement was reached on a final definition for clinical fistula healing: absence of any discharge symptoms, absence of abscess, infection, or inflammation, and no recurrence or persistence for a continuous period of at least six months. This definition was supported by 77% of participants. The preferred assessment modality was clinical assessment, defined as a combination of clinical examination and history taking, which reached 96% agreement. Initial voting on the minimum duration of healing did not result in a majority, with 3 months and 6 months receiving 26% and 57% of the vote, respectively. A subsequent head-to-head vote led to consensus for the six-month threshold, with 75% agreement.

##### Recurrence

Recurrence was not redefined as a separate outcome but was interpreted based on the agreed definition for clinical healing. It was considered present when symptoms returned following a documented phase of healing. MRI (where feasible) and clinical examination were confirmed as preferred modalities for assessing recurrence, supported by 85% of participants.

##### Radiological healing

For radiological healing, consensus was reached on a definition describing complete resolution of any visible fistula tract and inflammatory mass, with or without the presence of fibrosis within the tract. This definition was supported by 100% of participants. MRI was confirmed as the preferred assessment modality, with 88% agreement.

##### Development of additional fistula

Although no formal vote was taken on the definition of “development of an additional fistula,” participants agreed that this outcome should be defined as the appearance of a new, anatomically distinct tract. Clinical examination and MRI (where feasible) were endorsed as preferred methods of assessment, receiving 96% agreement.

##### Complications and re-interventions

For complications and reinterventions, participants agreed that complications should be classified using the Clavien-Dindo classification system (86%) and that the term “reintervention” should be limited to surgical and radiological procedures like percutaneous abscess drainage (83%), because the use of antibiotics, for example, was so common after surgery and not necessarily undertaken on a firm evidential basis. Combined clinical assessment and MRI (where feasible) were endorsed as the preferred assessment modality, supported by 94% of participants.

A summary of all clinician reported outcomes can be found in Table 2.

**Table 2:** Consensus meeting results: clinician-reported outcomes.

	Definition	Modality
Clinical fistula healing	Absence of any discharge symptoms, absence of abscess, infection, or inflammation, and no recurrence or persistence for a continuous period of at least six months	Clinical assessment, defined as a combination of clinical examination and history taking
Recurrence	Present when symptoms returned following a documented healing phase of 6 months	MRI (where feasible) and clinical assessment
Radiological healing	Complete resolution of any visible fistula tract and inflammatory mass, with or without the presence of fibrosis within the tract	MRI (where feasible)
Development of additional fistula	Appearance of a new, anatomically distinct tract	Clinical examination and MRI (where feasible)
Complications and reinterventions	Clavien-Dindo classification system The term “reintervention” should be limited to surgical and radiological procedures	Clinical assessment and MRI (where feasible)

#### Consensus meeting: patient-reported outcomes

##### Quality of life

The AF-QOL was selected following a two-stage vote. In the initial round, it received 68.4%, QoLAF-Q 26.3%, FIQL 5.3%, and FISI 0%. In a head-to-head vote between AF-QOL and QoLAF-Q, the AF-QOL was chosen with 81% agreement.

##### Incontinence

Initial voting showed a tie between Vaizey and Wexner (both 43%); FIQL received 14% and COREFO 0%. After a head-to-head vote, the Vaizey score was selected with 71% agreement.

During the meeting, there was also a separate vote on whether the currently available measurement instruments for incontinence are ideal or whether a new instrument should be developed. In this vote, 55% supported the notion that a fistula-specific incontinence scale should be created in the future.

##### Fistula symptoms

The AF-QOL was selected with 86% agreement. The QoLAF-Q received 14% of the vote, while the Perianal Fistula Disease Severity Scores (PAD) and PDAI both received 0%.

##### Psychological impact of treatment

The AF-QOL was selected with 90% agreement. The QoLAF-Q and HADS each received 5% of the vote.

##### Patient satisfaction

This segment of the meeting initiated a discussion about whether an instrument should or could be selected for patient satisfaction, or whether a new tool should be developed. During a further discussion on the essence of this outcome, it became clear that developing a new instrument was not necessary as it represented the value in patients having the ability to evaluate their own treatment experience and outcomes, rather than their healthcare provider or the healthcare system. This interpretation was corroborated and confirmed by the patient representative. Therefore, a vote was conducted, and a generic 1–10 Numerical Rating Scale (NRS) was selected with 83% agreement. The PSQ-18 received 11% of the vote, and the GS-PEQ 6%.

A summary of all patient-reported outcomes can be found in Table 3.

**Table 3:** Consensus Meeting Results: Patient-reported outcomes.

	Measurement instrument
Quality of life	Anal Fistula Quality of Life Scale (AF-QOL)
Incontinence	The St. Mark's incontinence score (Vaizey)
Fistula symptoms	Anal Fistula Quality of Life Scale (AF-QOL)
Psychological impact of treatment	Anal Fistula Quality of Life Scale (AF-QOL)
Patient satisfaction	Numerical Rating Scale (NRS)

##### Timepoints

**Fig. 5:**
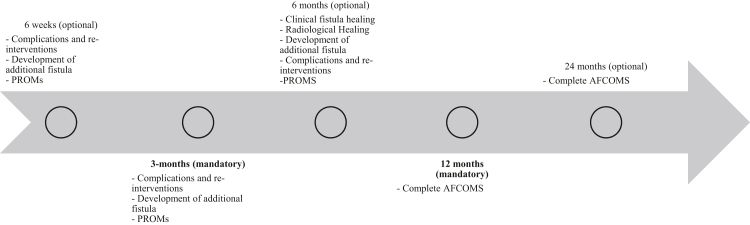
*Timeline of outcome measurements*.

For clinician-reported outcomes, assessments at 3- and 12-months post-treatment were deemed mandatory. Optional timepoints were proposed at 6 weeks, 6 months, and 24 months. This structure was supported by 90% of participants. The same structure was endorsed for patient-reported outcomes. Mandatory assessments were defined at 3 and 12 months, with 6 and 24 months considered optional. Consensus was reached for this timing framework, with 100% agreement. A timeline with required measurement instruments and outcomes at these timepoints can be seen in Fig. 5.

## Discussion

This study provides a structured Core Outcome Measurement Set (COMS) for cryptoglandular anal fistula, building on the previously defined Anal Fistula Core Outcome Set (AFCOS). The AFCOMS defines how and when each of the core outcomes should be measured, offering consensus-based definitions, measurement instruments, and timepoints applicable to clinical research.

This is the first COMS developed for cryptoglandular anal fistula and one of very few available within surgical research. Through a multi-phase process involving a scoping review, instrument appraisal, and a Delphi consensus procedure with international stakeholder participation, definitions, assessment modalities and measurement instruments were established for all outcomes included in the AFCOS. Definitions were finalised for all clinician-reported outcomes, including clinical and radiological healing, while appropriate instruments were selected for each patient-reported outcome, including quality of life, fistula symptoms, incontinence, psychological impact of treatment and patient satisfaction.

A major strength of this study lies in the composition of its international, multidisciplinary panel, which included healthcare professionals with substantial clinical and research experience in anal fistula surgery. The structured selection process for measurement instruments, which prioritised both validity, feasibility and patient involvement in development, supports the practical utility and global relevance of the recommendations. The use of structured instrument cards further enhanced transparency by providing participants with consistent, comprehensive summaries of psychometric properties and feasibility of each of the patient-reported outcome measurement instruments.

Patient involvement was broad but not without limitations. Patients participated in both Delphi rounds, and all patient-facing materials were adapted for clarity in English and Dutch. Several patients reported difficulties in interpreting measurement instrument properties, which caused a substantial drop out of patients in Round 1 of the Delphi survey, whereas in Round 2 all patients completed the survey. Given the limited patient representation, their input was heavily weighted within all discussions and particularly valuable in the selection of instruments for quality of life and incontinence, where their perspective closely aligned with patient preferences expressed earlier in the online Delphi rounds. After the Delphi survey, we also attempted to organise a dedicated patient meeting, but there was insufficient interest to attend, although patients were provided with the final consensus conclusions. Despite efforts to maximise diversity, most patient participants were Dutch- or English-speaking and predominantly Caucasian. Further translation of materials was not feasible within the scope of this study, limiting broader global inclusion. As COMS development is uncommon, transparent reporting of this limited patient participation is essential. Despite efforts to increase involvement, engagement was modest, underscoring challenges for future initiatives in niche conditions. Possible strategies include simplifying study materials, offering surveys in multiple languages, and providing short pre-round education to help patients appraise measurement instruments. Also, a dedicated patient society for cryptoglandular anal fistula patients could help to ensure that input is present in similar future works.

Another limitation to take into consideration is that not all selected instruments are available in all languages, and some assessment modalities, such as MRI, may not be available or feasible in all clinical settings. However, as AFCOMS is primarily intended for research rather than routine clinical practice, reproducibility and minimising inter-observer variability were prioritised. In this context, MRI was endorsed as the preferred imaging modality, consistent with the 2024 ESCP guideline on cryptoglandular anal fistula, due to its higher sensitivity and specificity compared with endoanal ultrasound. For this reason, MRI was recommended “where feasible,” providing a gold-standard reference for research, while recognising that feasibility and reimbursement will vary across healthcare systems. Local investigators are encouraged to assess practicality within their own context, but in research settings the required expertise for MRI interpretation can generally be expected. These limitations are common in international COS and COMS development and reflect broader disparities in healthcare resources. Feasibility information was therefore explicitly included within the instrument cards. Where possible, instruments available in multiple languages with minimal resource requirements were prioritised. For some outcomes, such as quality of life, disease-specific relevance was prioritised over broad availability. For example, the AF-QOL was selected over generic tools due to its stronger alignment with the lived experience of anal fistula, having been developed through qualitative patient interviews. Further work is needed to support translation and implementation, particularly in low-resource settings.

Looking ahead, implementation of the AFCOMS will require continued international collaboration. Future research should include longitudinal evaluation of current measurement practices against the AFCOMS and explore barriers and facilitators to its use in both research and clinical care. Application of the AFCOMS in trials and registries will promote standardisation, improve data comparability, and facilitate evidence synthesis. In clinical practice, the AFCOMS may support structured monitoring of outcomes such as long-term healing and broader patient-reported outcomes, ultimately contributing to more consistent and patient-centred care for individuals with cryptoglandular anal fistula.

The final Anal Fistula Core Outcome Measurement Set (AFCOMS) provides clear guidance on how to assess the clinician and patient-reported outcomes defined by the Anal Fistula Core Outcome Set (AFCOS), including standardised definitions and recommended measurement instruments. By reducing variability in outcome selection and measurement, AFCOMS supports consistent reporting across future studies on cryptoglandular anal fistula. This will enhance the comparability of findings, facilitate evidence synthesis, and strengthen the foundation for reliable meta-analyses and evidence-based guidelines. Ongoing international collaboration will be essential to ensure broad implementation and sustained impact in both research and clinical settings.

## Contributors

NT, SJ, MK, PT, and SB accessed and verified the underlying data. NT performed the data analyses, which were checked by SJ, MK, PT, and SB. The surveys were designed and created by NT, SJ, LM, MK, PT, and SB. NT chaired the consensus meeting and drafted the initial version of the manuscript, which was revised and approved by SJ, MK, PT, and SB. All consensus meeting collaborators reviewed and approved the final manuscript before submission. All authors had final responsibility for the decision to submit for publication.

## Data sharing statement

All de-identified data supporting the findings of this study, including Delphi round results and consensus meeting outcomes, are provided in the Supplementary Material. Additional information may be made available from the corresponding author upon reasonable request.

## Declaration of interests

PT declares receiving speaker fees and/or serving on advisory boards for Takeda, Johnson & Johnson, Tillots Pharma, Ferring, and Falk Pharma. NT, SJ, MK, LM, NI and SB declare no competing interests.

## Appendix A Supplementary data

Supplementary Clean

Collab authors
