# Supplementary material for: Cryptoglandular Anal Fistula Core Outcome Measurement Set (AFCOMS): standardised definitions and measurement instruments
Source: eClinicalMedicine. 2026 Feb 6;92:103745. doi: 10.1016/j.eclinm.2025.103745 (PMC12907631; doi:10.1016/j.eclinm.2025.103745)
Supplement: Supplementary Clean [file mmc1.pdf]

SUPPLEMENTARY

AFCOMS Consensus Meeting Collaborators

Figure S1. Frequency of definitional components for clinical healing, recurrence, and radiological healing across the literature (2008–2022)
Figure S2. Frequency of assessment modalities used for clinical healing, recurrence, and radiological healing (2008–2022)
Figure S3. Frequency of measurement instruments used for patient-reported outcomes (2008–2022)
Figure S4. Demographics of Delphi survey participants across both rounds
Figure S5. Geographic distribution of Delphi survey participants
Figure S6. Delphi survey results: Clinician-reported outcomes, definitions
Figure S7. Delphi survey results: Clinician-reported outcomes, assessment modalities
Figure S8. Delphi survey Round 1 results: Patient-reported outcomes, measurement instruments
Figure S9. Delphi survey Round 2 results: Patient-reported outcomes, measurement instruments
Figure S10. Summary of instrument properties

Appendix

Search for validation studies using the COSMIN-developed filter

AFCOMS Consensus Meeting Collaborators

First name	Last name	Affiliation
Ademola	Adeyeye	Colorectal Surgery, King's College London, London, United Kingdom
Andrea Marco	Tamburini	Department of General Surgery, IRCCS San Raffaele Scientific Institute, Milan, Italy
Angelo Alessandro	Marra	Proctology and Pelvic Floor Surgery Unit, Ospedale Isola Tiberina, Gemelli Isola, Rome, Italy
Anthony	Lin	Wellington Colorectal Surgery, Wellington, New Zealand
Arda	Isik	Department of Surgery, Faculty of Medicine, Istanbul Medeniyet University, Istanbul, Turkey
Eleni	Andriopoulou	Brighton and Sussex University Hospitals NHS Trust, Brighton, United Kingdom
Fatima	Senra	Department of General and Colorectal Surgery, Weston General Hospital, Weston-super-Mare, United Kingdom
Flavia	Alexandre	Colorectal Unit, Department of General Surgery, Hospital Universitario Austral, Buenos Aires, Argentina
Francesco	Pata	Department of Surgical Sciences, University of Calabria, Rende, Italy
Gaetano	Gallo	Vita-Salute San Raffaele University, Colorectal and Pelvic Floor Surgery Unit, Milan, Italy
Gabriele	Bislenghi	Department of Surgery, University Hospitals Leuven, Leuven, Belgium
Jasper	Stijns	Department of Surgery, Universitair Ziekenhuis Brussel, Brussels, Belgium
Jesus	Lopez-Alcalde	Institute for Complementary and Integrative Medicine, University Hospital Zurich and University of Zurich, Zurich, Switzerland

First name	Last name	Affiliation
Johannes	Jongen	Department of General, Visceral, Vascular and Transplant Surgery, University Hospital Essen, Essen, Germany
Lilli	Lundby	Department of Surgery, Aarhus University Hospital, Aarhus, Denmark
Lillian	Reza	Robin Phillips Fistula Research Unit, St Mark's Hospital, Imperial College London, London, United Kingdom
Martijn	Gosselink	Department of Colorectal Surgery, Dr. Horacio E. Oduber Hospital, Oranjestad, Aruba
Peter	Ambe	Department of Surgery, Witten/Herdecke University, Witten, Germany
Philip	Lung	St Mark's Hospital, London, United Kingdom
Raimund	Strouhal	Department of Surgery, Salzkammergut Klinikum, Austria
Ricardo	Rocha	Department of General Surgery, Hospital Professor Doutor Fernando Fonseca, Amadora, Portugal
Giulio Aniello	Santoro	Department of Surgery, Treviso Regional Hospital (ULSS 2 Marca Trevigiana), Treviso, Italy
Stephen	Ward	Department of Colorectal Surgery, University Hospitals Birmingham NHS Foundation Trust, Birmingham, United Kingdom

Figure S1. *Frequency of definitional components for clinical healing, recurrence, and radiological healing across the literature (2008–2022)*

**Definitional Components "Clinical Fistula Healing"**	**Times used**<br>January 2008 – August 2022<br>Total / (updated search)
Absence of symptoms	81 (11)
Closure of the external fistula opening	75 (14)
Absence of abscess or infection or inflammation or sepsis	32 (5)
Closure of the (surgical) wound	25 (1)
Closure of the internal fistula opening	19 (4)
Closure of the fistula tract	14
Absence of recurrence or persistence or treatment failure	9 (1)
No additional intervention required	8
Absence of anal sphincter injury	1
**Definitional Components "Recurrence"**	**Times used**<br>January 2008 – August 2022<br>Total / (updated search)
Persistence or recurrence of symptoms	30 (9)
Reappearance of the fistula after healing	20 (4)
Persistence or reappearance of the external fistula opening	20 (7)
Absence of wound healing	11 (3)
Abscess or infection	7 (1)
Absence of fistula closure or persistence	6
Additional fistula	5 (3)
Non-healing fistula	5 (2)
Additional intervention required	4 (1)
**Definitional Components "Radiological Healing"**	**Times used**<br>May 2020 - August 2022<br>Total / (updated search)
Absence of inflammatory mass	2 (2)
Hyperintensity of the tract on T2-weighted images	1 (1)
Hyperintensity of the tract on post-contrast images	1 (1)
Fibrosis of the tract	1 (1)
Granulation of the tract	1 (1)

Note. This figure presents definitional components used in the literature to describe clinical healing, recurrence, and radiological healing. Frequencies refer to the number of studies using each component between January 2008 and August 2022. Numbers in brackets indicate results from the updated search (January 2020– August 2022).

Figure S2. *Frequency of assessment modalities used for clinical healing, recurrence, and radiological healing across the literature (2008–2022)*

**Assessment Modalities "Clinical Fistula Healing"**	**Times used**<br>January 2008 – August 2022<br>Total / (updated search)
Clinical examination, including digital rectal examination	106 (18)
(Telephone) interview	17 (1)
MRI	11 (2)
Medical record review	9 (2)
Anoscopy or proctoscopy or rectoscopy	8 (1)
(3D) endoanal ultrasound	7
(Un)specified questionnaire	6
Anal endosonography	5 (4)
Digital photograph of the external fistula opening	2
Transanal ultrasound	1
Examination under anaesthetic	1
Perianal Fıstula Disease Severity Scores	1 (1)
Perianal disease activity index (PDAI)	1 (1)
**Assessment Modalities "Recurrence"**	**Times used**<br>January 2008 – August 2022<br>Total / (updated search)
Clinical examination	47 (4)
Medical record review	9 (3)
MRI	8 (2)
(Telephone) interview	7
Endorectal ultrasound	4 (2)
(Un)specified questionnaire	3
Anoscopy or proctoscopy	2
3D endoanal ultrasound	1
Anal endosonography	1
Patient-reported	1
**Assessment Modalities "Radiological Healing"**	**Times used**<br>May 2020 - August 2022<br>Total / (updated search)
MRI	4 (4)
(3D) endoanal ultrasound	2 (2)

Note. This figure presents assessment modalities used in the literature to describe clinical healing, recurrence, and radiological healing. Frequencies refer to the number of studies using each component between January 2008 and August 2022. Numbers in brackets indicate results from the updated search (January 2020– August 2022).

Figure S3. *Frequency of measurement instruments used for quality of life, incontinence, fistula symptoms, psychological impact of treatment and patient satisfaction across the literature (2008–2022)*

**Measurement Instrument for "Quality of life"**	**Times used**<br>January 2008 – August 2022<br>Total / (updated search)
Fecal Incontinence Quality of Life Scale	9 (3)
Short Form-36 health survey (SF-36)	8 (2)
EQ-5D	5 (1)
Cleveland global quality of life	3 (1)
Short Form-12 health survey (SF-12)	2
Gastrointestinal Quality of Life Index	2
(Un)specified questionnaire	2
Quality of Life in patients with Anal Fistula Questionnaire (QoLAF-Q)	1 (1)
Quality of Life Scale	1
Visual Analogue Scale (VAS)	1
Fecal Incontinence Severity Index	1
**Measurement Instrument for "Incontinence"**	**Times used**<br>January 2008 – August 2022<br>Total / (updated search)
Wexner Cleveland Clinic Florida incontinence score	69 (21)
Vaizey incontinence score	13 (4)
Patient-reported	10 (1)
Fecal Incontinence Quality of Life Scale	7 (1)
Anorectal manometry	6 (1)
Medical record review	5 (3)
Endoanal ultrasound	5 (1)
(Un)specified questionnaire	5
Specified grading system	4 (1)
Clinical examination, including digital rectal examination	4 (1)
Fecal Incontinence Severity Index	3 (2)
Colorectal functional outcome questionnaire	3
(Telephone) interview	3
German Society of Coloproctology score	1
Williams grade	1

**Measurement Instrument for "Fistula Symptoms"**	**Times used**<br>May 2020 - August 2022<br>Total / (updated search)
Perianal Fıstula Disease Severity Scores	1 (1)
Perianal Disease activity index (PDAI)	1 (1)
**Measurement Instrument for "Psychological impact of treatment"**	**Times used**<br>May 2020 - August 2022<br>Total / (updated search)
Hospital Anxiety and Depression Scale (HADS)	1 (1)

**Measurement Instrument for "Patient Satisfaction"**	**Times used**

	May 2020 - August 2022<br>Total / (updated search)
Numerical rating scale (1-10)	3 (3)
PSQ-18	1 (1)
GS-PEQ	1 (1)

Note. This figure presents measurement instruments used in the literature to describe quality of life, incontinence, fistula symptoms, psychological impact of treatment and patient satisfaction. Frequencies refer to the number of studies using each component between January 2008 and August 2022. Numbers in brackets indicate results from the updated search (January 2020–August 2022).

Figure S4. *Demographics Delphi Survey Rounds*

	**Survey Round 1**	**Survey Round 2**
	Healthcare professionals<br>N = 95 (Percentage)	Healthcare professionals<br>N = 69 (Percentage)
**Gender**		
Male	70 (74)	52 (75)
Female	25 (26)	17 (25)
**Years of practice**		
0-5	24 (25)	17 (25)
6-10	21 (22)	14 (20)
11-20	29 (31)	21 (30)
>20	21 (22)	17 (25)
**Self-reported expertise**		
Gastroenterologist/ proctologist	6 (6)	7 (10)
General colorectal surgeon	38 (40)	22 (32)
Colorectal surgeon with interest in fistula	49 (52)	38 (55)
Specialist GI radiologist	2 (2)	2 (3)
**Volume of patients treated/seen annually**		
0-20	17 (18)	8 (12)
21-50	40 (42)	31 (45)
51-100	27 (28)	21 (30)
>100	11 (12)	9 (13)
**Work setting**		
General hospital/teaching hospital	53 (56)	38 (55)
Tertiary referral centre	32 (34)	25 (36)
Private Hospital	10 (11)	6 (9)

	**Survey Round 1**	**Survey Round 2**
	Patients<br>N = 15 (Percentage)	Patients<br>N = 7 (Percentage)
**Gender**		
Male	4 (27)	1 (14)
Female	11 (73)	6 (86)
**Age**		
21-30	3 (20)	1 (14)
31-40	6 (40)	5 (71)
41-50	5 (33)	1 (14)
51-60	1 (7)	0 (0)
**Ethnicity**		
White/ Caucasian	13 (87)	6 (86)
Black	1 (7)	0 (0)
Other	1 (7)	1 (14)

**Duration of fistula**		
0-6 months	3 (20)	3 (43)
7-12 months	4 (27)	1 (14)
>12 months	8 (53)	3 (43)
**Current fistula status**		
Current symptomatic fistula, not had treatment yet	2 (13)	2 (29)
Previously had a fistula that has been treated and healed, or given me no symptoms	5 (33)	1 (14)
Undergone treatment but fistula still active	8 (53)	4 (57)
**Hospital setting for treatment (select all that apply)**		
DGH	5 (33)	3 (43)
Tertiary referral centre	9 (60)	4 (57)
Private Hospital	3 (20)	0 (0)

Figure S5. *Demographics Delphi Rounds Countries of Residence*

	**All participants Round 1**<br>N = 110 / (15 Patients)	**All participants Round 2**<br>N = 76 / (7 Patients)
**Africa**		
Egypt	2	1
Nigeria	1	1
Tunisia	1	1
**Asia**		
India	1	1
Malaysia	1	1
**Europe**		
Austria	2	1
Belgium	2	2
Bulgaria	2	1
Czech Republic	3	1
Denmark	1	1
Finland	1	0
France	1	0
Germany	5	2
Greece	3	1
Italy	16	11
Netherlands	12 (7)	6 (3)
Norway	1	1
Poland	1	1
Portugal	3	3
Russia	4	4
Slovenia	1	1
Spain	6	6
Sweden	1 (1)	1 (1)
Turkey	4	4
United Kingdom	26 (4)	18 (2)
**North America**		
Aruba	1	1
Canada	1 (1)	1 (1)
US	2 (2)	0
**Oceania**		
Australia	1	0
New Zealand	1	1
**South America**		
Argentina	1	1
Chile	2	2

Figure S6. *Delphi Survey Results: Clinician Reported Outcomes, Definitions*

**Clinical fistula healing**	**Survey Round 1** *GRADE 6-9 scores*	**Survey Round 2** *GRADE 6-9 scores*
Absence of symptoms	79%	84%
Absence of abscess or infection or inflammation or sepsis	76%	76%
Absence of recurrence or persistence or treatment failure	67%	72%
Closure of the internal fistula opening	55%	53%
Closure of the external fistula opening	54%	64%
Closure of the (surgical) wound	47%	52%
Closure of the fistula tract	44%	42%
No additional intervention required	36%	26%
Absence of anal sphincter injury	17%	10%

**Radiological healing**	**Survey Round 1** *GRADE 6-9 scores*	**Survey Round 2** *GRADE 6-9 scores*
Absence of inflammatory mass	75%	80%
Fibrosis of the tract	62%	73%
Hyperintensity of the tract on T2-weighted images	61%	68%
Hyperintensity of the tract on post-contrast images	30%	20%
Granulation of the tract	7%	9%

Figure S7. *Delphi Survey Results: Clinician Reported Outcomes, Modalities*

**Clinical fistula healing**	**Survey Round 1** *Healthcare professional % GRADE 6-9 / Patient median GRADE score*	**Survey Round 2** *Healthcare professional % GRADE 6-9 / Patient median GRADE score*
Clinical examination	96% / 8	95% / 8
History taking	70% / 7	63% / 8
Examination under anaesthetic	32% / 5	26% / 5

**Radiological healing**	**Survey Round 1** *Healthcare professional % GRADE 6-9 / Patient median GRADE score*	**Survey Round 2** *Healthcare professional % GRADE 6-9 / Patient median GRADE score*
MRI	96%/ 8	95% / 8
Ultrasound (endoanal)	55%/ 5	67% / 5

**Recurrence**	**Survey Round 1** *Healthcare professional % GRADE 6-9 / Patient median GRADE score*	**Survey Round 2** *Healthcare professional % GRADE 6-9 / Patient median GRADE score*
Clinical examination	96%/ 8	92%/ 8
MRI	79%/ 7	83%/ 7
History taking	71%/ 8	63%/ 8
Ultrasound (endoanal)	55%/ 5	63%/ 5
Examination under anaesthetic	32%/ 5	28%/ 5
Chart review	9%/ 5	5%/ 5

**Development of additional fistula**	**Survey Round 1** *Healthcare professional % GRADE 6-9 / Patient median GRADE score*	**Survey Round 2** *Healthcare professional % GRADE 6-9 / Patient median GRADE score*
MRI	91%/ 6	95%/ 7
Clinical examination	89%/ 6	86%/ 7
History taking	63%/ 7	62%/ 7
Ultrasound (endoanal)	60%/ 6	72%/ 6
Examination under anaesthetic	56%/ 6	48%/ 6
Chart review	15%/ 5	8%/ 5

**Complications and reinterventions**	**Survey Round 1** *Healthcare professional % GRADE 6-9 / Patient median GRADE score*	**Survey Round 2** *Healthcare professional % GRADE 6-9 / Patient median GRADE score*
Clinical examination	93%/ 6	88%/ 8
History taking	81%/ 7	85%/ 7
MRI	67%/ 7	74%/ 8
Examination under anaesthetic	55%/ 7	46%/ 6
Ultrasound (endoanal)	47%/ 6	42%/ 6
Chart review	29%/ 5	26%/ 5

Figure S8. *Delphi Survey Round 1 Results: Patient Reported Outcomes, Measurement instruments*

**Quality of Life Scales**	*Overall group % GRADE 7-9*	*Healthcare professional % GRADE 7-9*	*Patients' Median GRADE*
FISI	64,9%	67,2%	7
QoLAF-Q	75,7%	73,4%	8
FIQL	63,6%	60,6%	8
SF-36	32,9%	27,3%	6
EQ-5D	27,0%	26,6%	6
SF-12	31,1%	31,6%	7
GIQL	20,5%	17,5%	5
AF-QOL	N/A	N/A	N/A

**Incontinence**	*Overall group % GRADE 7-9*	*Healthcare professional % GRADE 7-9*	*Patients' Median GRADE*
Wexner	75,7%	78,1%	7
Vaizey	65,7%	67,2%	6
FIQL	64,7%	61,3%	8
COREFO	33,3%	27,6%	8

**Fistula Symptoms**	*Overall group % GRADE 7-9*	*Healthcare professional % GRADE 7-9*	*Patients' Median GRADE*
PAD	59,7%	57,4%	7
PDAI	57,6%	54,1%	8

**Psychological impact of treatment**	*Overall group % GRADE 7-9*	*Healthcare professional % GRADE 7-9*	*Patients' Median GRADE*
HADS	38,5%	33,9%	8

**Patient satisfaction**	*Overall group % GRADE 7-9*	*Healthcare professional % GRADE 7-9*	*Patients' Median GRADE*
GS-PEQ	32,8%	29,3%	7
NRS	64,6%	66,1%	6
PSY-Q	54,7%	51,7%	7

Figure S9. *Delphi Survey Round 2 Results: Patient Reported Outcomes, Measurement instruments*

**Quality of Life Scales**	***Overall group % GRADE 7-9***	***Healthcare professional % GRADE 7-9***	***Patients' Median GRADE***	***If patient input was decisive for inclusion in consensus meeting***
FISI	73,7%	73,7%	8	Included
QoLAF-Q	76,3%	76,3%	7	Included
FIQL	57,9%	57,9%	7	Included (patient input decisive)
SF-36	31,6%	31,6%	6	Excluded
EQ-5D	34,2%	34,2%	6	Excluded
SF-12	23,7%	23,7%	6	Excluded
GIQL	18,4%	18,4%	5	Excluded
AF-QOL	76,4%	76,3%	8	Included

**Incontinence**	***Overall group % GRADE 7-9***	***Healthcare professional % GRADE 7-9***	***Patients' Median GRADE***	***If patient input was decisive for inclusion in consensus meeting***
Wexner	76,3%	75,4%	7	Included
Vaizey	78,9%	78,3%	7	Included
FIQL	50,0%	46,4%	8	Included (patient input decisive)
COREFO	35,5%	31,9%	7	Included (patient input decisive)

**Fistula Symptoms**	***Overall group % GRADE 7-9***	***Healthcare professional % GRADE 7-9***	***Patients' Median GRADE***	***If patient input was decisive for inclusion in consensus meeting***
AF-QOL	*84,1%*	*82%*	*8*	Included
PAD	56,8%	55,2%	6	Excluded
PDAI	58,1%	56,7%	7	Included (patient input decisive)

**Psychological impact of treatment**	***Overall group % GRADE 7-9***	***Healthcare professional % GRADE 7-9***	***Patients' Median GRADE***	***If patient input was decisive for inclusion in consensus meeting***
AF-QOL	*68,1%*	*66%*	*8*	Included (patient input decisive)
HADS	48,6%	46,9%	7	Included (patient input decisive)

**Patient satisfaction**	***Overall group % GRADE 7-9***	***Healthcare professional % GRADE 7-9***	***Patients' Median GRADE***	***If patient input was decisive for inclusion in consensus meeting***
GS-PEQ	34,3%	31,3%	6	Excluded
NRS	68,6%	71,9%	6	Excluded
PSY-Q	54,3%	53,1%	6	Excluded

Figure S10. *Summary of instrument properties*

**Instrument**	**Acronym**	**Domain(s)**	**Items**	**Est. time**	**Use in cryptoglandular anal fistula studies (2008–2022)**	**Validation in cryptoglandular anal fistula population**	**Highest COSMIN rating**
Perianal Fistula Disease Severity Scores	PAF / PAD severity	Clinical fistula healing	1	1–2 min	1	No	No evaluation
Perianal Disease Activity Index	PDAI	Healing	5	3–5 min	1	No	No evaluation
Fecal Incontinence Quality of Life Scale	FIQL (Rockwood)	QoL; Incontinence	29	Not stated	9 (QoL) + 7 (incontinence)	No	No evaluation
Fecal Incontinence Severity Index	FISI	Incontinence; QoL	4	< 1 min	1 (QoL context)	No	No evaluation
Quality of Life Anal Fistula Questionnaire	QoLAF-Q	Fistula symptoms; QoL	14	Not stated	1	Yes, limited	Content validity rated (very) low [Iqbal 2022]
Gastrointestinal Quality of Life Index	GIQLI	QoL	36	~5 min	1	Yes, limited	Content validity rated (very) low [Iqbal 2022]
36-Item Short Form Health Survey	SF-36	QoL	36	7–8 min	8	No	No evaluation
12-Item Short Form Health Survey	SF-12	QoL	12	4–5 min	2	No	No evaluation
EuroQol	EQ-5D	QoL	6	5–10 min	5	No	No evaluation
Wexner Cleveland Clinic Score	Wexner	Incontinence	5	< 5 min	69	No in cryptoglandular anal fistula	No evaluation
Vaizey Incontinence Score	Vaizey	Incontinence	7	< 5 min	11	No in cryptoglandular anal fistula	No evaluation
Colorectal Functional Outcome Questionnaire	COREFO	Incontinence; Quality of life	27	10–15 min	3	No	No evaluation
Anal Fistula Quality of Life Scale	AF-QoL	QoL, Psychological impact, Fistula Symptoms	22	5–10 min	Developed after 2022	Yes, development study in cryptoglandular anal fistula	Initial validation reported in development study
Hospital Anxiety and Depression Scale	HADS	Psychological impact	14	5–10 min	1	No	No evaluation

APPENDIX

Search for validation studies using the COSMIN-developed filter per measurement instrument

Measurement instrument	Hits / Eligible
Perianal Fistula Disease Severity Scores	26 / 0
Perianal disease activity index (PDAI)	17 / 0
Wexner Cleveland Clinic Florida incontinence score	98 / 0
Vaizey incontinence score	14 / 0
Fecal Incontinence Quality of Life Scale	23 / 0
Colorectal functional outcome questionnaire	29 / 0
St. Mark's Incontinence Score (SMIS)	10 / 0
German Society of Coloproctology Score	1 / 0
Williams grade	3 / 0
Short Form-36 health survey (SF-36)	14 / 0
EQ-5D	3 / 0
Cleveland global quality of life	6 / 0
Visual Analogue Scale (VAS)	60 / 0
Short Form-12 health survey (SF-12)	9 / 0
Fecal Incontinence Severity Index (FISI)	52 / 0
Gastrointestinal Quality of Life Index (GIQLI)	34 / 1<br>10.1046/j.1365-2168.1998.00958.x
Quality of Life in patients with Anal Fistula Questionnaire (QoLAF-Q)	85 / 2<br>10.1097/DCR.0000000000000877<br>10.3390/clinpract12040066.

Filter
Including:
(instrumentation[sh] OR methods[sh] OR “Validation Studies”[pt] OR “Comparative Study”[pt] OR “psychometrics”[MeSH] OR psychometr*[tiab] OR clinimetr*[tw] OR clinometr*[tw] OR “outcome assessment (health care)”[MeSH] OR “outcome assessment”[tiab] OR “outcome measure*”[tw] OR “observer variation”[MeSH] OR “observer variation”[tiab] OR “Health Status Indicators”[Mesh] OR “reproducibility of results”[MeSH] OR reproducib*[tiab] OR “discriminant analysis”[MeSH] OR reliab*[tiab] OR unreliab*[tiab] OR valid*[tiab] OR “coefficient of variation”[tiab] OR coefficient[tiab] OR homogeneity[tiab] OR homogeneous[tiab] OR “internal consistency”[tiab] OR (cronbach*[tiab] AND (alpha[tiab] OR alphas[tiab])) OR (item[tiab] AND (correlation*[tiab] OR selection*[tiab] OR reduction*[tiab])) OR agreement[tw] OR precision[tw] OR imprecision[tw] OR “precise values”[tw] OR test-retest[tiab] OR (test[tiab] AND retest[tiab]) OR (reliab*[tiab] AND (test[tiab] OR retest[tiab])) OR stability[tiab] OR interrater[tiab] OR inter-rater[tiab] OR intrarater[tiab] OR intra-rater[tiab] OR intertester[tiab] OR inter-tester[tiab] OR intratester[tiab] OR intra-tester[tiab] OR interobserver[tiab] OR inter-observer[tiab] OR intraobserver[tiab] OR intra-observer[tiab] OR intertechnician[tiab] OR inter-technician[tiab] OR intratechnician[tiab] OR intra-technician[tiab] OR interexaminer[tiab] OR inter-examiner[tiab] OR intraexaminer[tiab] OR intra-examiner[tiab] OR interassay[tiab] OR inter-assay[tiab] OR intraassay[tiab] OR intra-assay[tiab] OR interindividual[tiab] OR inter-individual[tiab] OR intraindividual[tiab] OR intra-individual[tiab] OR interparticipant[tiab] OR inter-participant[tiab] OR intraparticipant[tiab] OR intra-participant[tiab] OR kappa[tiab] OR kappa’s[tiab] OR kappas[tiab] OR repeatab*[tw] OR ((replicab*[tw] OR repeated[tw]) AND (measure[tw] OR measures[tw] OR findings[tw] OR result[tw] OR results[tw] OR test[tw] OR tests[tw])) OR generaliza*[tiab] OR generalisa*[tiab] OR concordance[tiab] OR (intraclass[tiab] AND correlation*[tiab]) OR discriminative[tiab] OR

“known group”[tiab] OR “factor analysis”[tiab] OR “factor analyses”[tiab] OR “factor structure”[tiab] OR “factor structures”[tiab] OR dimension*[tiab] OR subscale*[tiab] OR (multitrait[tiab] AND scaling[tiab] AND (analysis[tiab] OR analyses[tiab])) OR “item discriminant”[tiab] OR “interscale correlation*”[tiab] OR error[tiab] OR errors[tiab] OR “individual variability”[tiab] OR “interval variability”[tiab] OR “rate variability”[tiab] OR (variability[tiab] AND (analysis[tiab] OR values[tiab])) OR (uncertainty[tiab] AND (measurement[tiab] OR measuring[tiab])) OR “standard error of measurement”[tiab] OR sensitiv*[tiab] OR responsive*[tiab] OR (limit[tiab] AND detection[tiab]) OR “minimal detectable concentration”[tiab] OR interpretab*[tiab] OR ((minimal[tiab] OR minimally[tiab] OR clinical[tiab] OR clinically[tiab]) AND (important[tiab] OR significant[tiab] OR detectable[tiab]) AND (change[tiab] OR difference[tiab])) OR (small*[tiab] AND (real[tiab] OR detectable[tiab]) AND (change[tiab] OR difference[tiab])) OR “meaningful change”[tiab] OR “ceiling effect”[tiab] OR “floor effect”[tiab] OR “Item response model”[tiab] OR IRT[tiab] OR Rasch[tiab] OR “Differential item functioning”[tiab] OR DIF[tiab] OR “computer adaptive testing”[tiab] OR “item bank”[tiab] OR “cross-cultural equivalence”[tiab])
Excluding:
(“addresses”[Publication Type] OR “biography”[Publication Type] OR “case reports”[Publication Type] OR “comment”[Publication Type] OR “directory”[Publication Type] OR “editorial”[Publication Type] OR “festschrift”[Publication Type] OR “interview”[Publication Type] OR “lectures”[Publication Type] OR “legal cases”[Publication Type] OR “legislation”[Publication Type] OR “letter”[Publication Type] OR “news”[Publication Type] OR “newspaper article”[Publication Type] OR “patient education handout”[Publication Type] OR “popular works”[Publication Type] OR “congresses”[Publication Type] OR “consensus development conference”[Publication Type] OR “consensus development conference, nih”[Publication Type] OR “practice guideline”[Publication Type]) NOT (“animals”[MeSH Terms] NOT “humans”[MeSH Terms])

Perianal Fıstula Disease Severity Scores
Hits: 26
Eligible: 0
((("Perianal Fıstula Disease Severity Scores") AND (anal fistula)) AND ((instrumentation[sh] OR methods[sh] OR "Validation Studies"[pt] OR "Comparative Study"[pt] OR "psychometrics"[MeSH] OR psychometr*[tiab] OR clinimetr*[tw] OR clinometr*[tw] OR "outcome assessment (health care)"[MeSH] OR "outcome assessment"[tiab] OR "outcome measure*"[tw] OR "observer variation"[MeSH] OR "observer variation"[tiab] OR "Health Status Indicators"[Mesh] OR "reproducibility of results"[MeSH] OR reproducib*[tiab] OR "discriminant analysis"[MeSH] OR reliab*[tiab] OR unreliab*[tiab] OR valid*[tiab] OR "coefficient of variation"[tiab] OR coefficient[tiab] OR homogeneity[tiab] OR homogeneous[tiab] OR "internal consistency"[tiab] OR (cronbach*[tiab] AND (alpha[tiab] OR alphas[tiab])) OR (item[tiab] AND (correlation*[tiab] OR selection*[tiab] OR reduction*[tiab])) OR agreement[tw] OR precision[tw] OR imprecision[tw] OR "precise values"[tw] OR test-retest[tiab] OR (test[tiab] AND retest[tiab]) OR (reliab*[tiab] AND (test[tiab] OR retest[tiab])) OR stability[tiab] OR interrater[tiab] OR inter-rater[tiab] OR intrarater[tiab] OR intra-rater[tiab] OR intertester[tiab] OR inter-tester[tiab] OR intratester[tiab] OR intra-tester[tiab] OR interobserver[tiab] OR inter-observer[tiab] OR intraobserver[tiab] OR intra-observer[tiab] OR intertechnician[tiab] OR inter-technician[tiab] OR intratechnician[tiab] OR intra-technician[tiab] OR interexaminer[tiab] OR inter-examiner[tiab] OR intraexaminer[tiab] OR intra-examiner[tiab] OR interassay[tiab] OR inter-assay[tiab] OR intraassay[tiab] OR intra-assay[tiab] OR interindividual[tiab] OR inter-individual[tiab] OR intraindividual[tiab] OR intra-individual[tiab] OR interparticipant[tiab] OR inter-participant[tiab] OR intraparticipant[tiab] OR intra-participant[tiab] OR kappa[tiab] OR kappa’s[tiab] OR kappas[tiab] OR repeatab*[tw] OR ((replicab*[tw] OR repeated[tw]) AND (measure[tw] OR measures[tw] OR findings[tw] OR result[tw] OR results[tw] OR test[tw] OR tests[tw])) OR generaliza*[tiab] OR generalisa*[tiab] OR concordance[tiab] OR (intraclass[tiab] AND correlation*[tiab]) OR discriminative[tiab] OR "known group"[tiab] OR "factor analysis"[tiab] OR "factor analyses"[tiab] OR "factor structure"[tiab] OR "factor structures"[tiab] OR dimension*[tiab] OR subscale*[tiab] OR (multitrait[tiab] AND scaling[tiab] AND (analysis[tiab] OR analyses[tiab])) OR "item discriminant"[tiab] OR "interscale correlation*"[tiab] OR error[tiab] OR errors[tiab] OR "individual variability"[tiab] OR "interval variability"[tiab] OR "rate variability"[tiab] OR (variability[tiab] AND (analysis[tiab] OR values[tiab])) OR (uncertainty[tiab] AND (measurement[tiab] OR measuring[tiab])) OR "standard error of measurement"[tiab] OR sensitiv*[tiab] OR responsive*[tiab] OR (limit[tiab] AND detection[tiab]) OR "minimal detectable concentration"[tiab] OR interpretab*[tiab] OR ((minimal[tiab] OR minimally[tiab] OR clinical[tiab] OR clinically[tiab]) AND (important[tiab] OR significant[tiab] OR detectable[tiab]) AND (change[tiab] OR difference[tiab])) OR (small*[tiab] AND (real[tiab] OR detectable[tiab]) AND (change[tiab] OR difference[tiab])) OR "meaningful change"[tiab] OR "ceiling effect"[tiab] OR "floor effect"[tiab] OR "Item response model"[tiab] OR IRT[tiab] OR Rasch[tiab] OR "Differential item functioning"[tiab] OR DIF[tiab] OR "computer adaptive testing"[tiab]

OR "item bank"[tiab] OR "cross-cultural equivalence"[tiab]))) NOT (("addresses"[Publication Type] OR "biography"[Publication Type] OR "case reports"[Publication Type] OR "comment"[Publication Type] OR "directory"[Publication Type] OR "editorial"[Publication Type] OR "festschrift"[Publication Type] OR "interview"[Publication Type] OR "lectures"[Publication Type] OR "legal cases"[Publication Type] OR "legislation"[Publication Type] OR "letter"[Publication Type] OR "news"[Publication Type] OR "newspaper article"[Publication Type] OR "patient education handout"[Publication Type] OR "popular works"[Publication Type] OR "congresses"[Publication Type] OR "consensus development conference"[Publication Type] OR "consensus development conference, nih"[Publication Type] OR "practice guideline"[Publication Type]) NOT ("animals"[MeSH Terms] NOT "humans"[MeSH Terms]))

Perianal disease activity index (PDAI)
Hits: 17
Eligible: 0
(((("Perianal disease activity index") OR (PDAI)) AND (anal fistula)) AND ((instrumentation[sh] OR methods[sh] OR "Validation Studies"[pt] OR "Comparative Study"[pt] OR "psychometrics"[MeSH] OR psychometr*[tiab] OR clinimetr*[tw] OR clinometr*[tw] OR "outcome assessment (health care)"[MeSH] OR "outcome assessment"[tiab] OR "outcome measure*"[tw] OR "observer variation"[MeSH] OR "observer variation"[tiab] OR "Health Status Indicators"[Mesh] OR "reproducibility of results"[MeSH] OR reproducib*[tiab] OR "discriminant analysis"[MeSH] OR reliab*[tiab] OR unreliab*[tiab] OR valid*[tiab] OR "coefficient of variation"[tiab] OR coefficient[tiab] OR homogeneity[tiab] OR homogeneous[tiab] OR "internal consistency"[tiab] OR (cronbach*[tiab] AND (alpha[tiab] OR alphas[tiab])) OR (item[tiab] AND (correlation*[tiab] OR selection*[tiab] OR reduction*[tiab])) OR agreement[tw] OR precision[tw] OR imprecision[tw] OR "precise values"[tw] OR test-retest[tiab] OR (test[tiab] AND retest[tiab]) OR (reliab*[tiab] AND (test[tiab] OR retest[tiab])) OR stability[tiab] OR interrater[tiab] OR inter-rater[tiab] OR intrarater[tiab] OR intra-rater[tiab] OR intertester[tiab] OR inter-tester[tiab] OR intratester[tiab] OR intra-tester[tiab] OR interobserver[tiab] OR inter-observer[tiab] OR intraobserver[tiab] OR intra-observer[tiab] OR intertechnician[tiab] OR inter-technician[tiab] OR intratechnician[tiab] OR intra-technician[tiab] OR interexaminer[tiab] OR inter-examiner[tiab] OR intraexaminer[tiab] OR intra-examiner[tiab] OR interassay[tiab] OR inter-assay[tiab] OR intraassay[tiab] OR intra-assay[tiab] OR interindividual[tiab] OR inter-individual[tiab] OR intraindividual[tiab] OR intra-individual[tiab] OR interparticipant[tiab] OR inter-participant[tiab] OR intraparticipant[tiab] OR intra-participant[tiab] OR kappa[tiab] OR kappa’s[tiab] OR kappas[tiab] OR repeatab*[tw] OR ((replicab*[tw] OR repeated[tw]) AND (measure[tw] OR measures[tw] OR findings[tw] OR result[tw] OR results[tw] OR test[tw] OR tests[tw])) OR generaliza*[tiab] OR generalisa*[tiab] OR concordance[tiab] OR (intraclass[tiab] AND correlation*[tiab]) OR discriminative[tiab] OR "known group"[tiab] OR "factor analysis"[tiab] OR "factor analyses"[tiab] OR "factor structure"[tiab] OR "factor structures"[tiab] OR dimension*[tiab] OR subscale*[tiab] OR (multitrait[tiab] AND scaling[tiab] AND (analysis[tiab] OR analyses[tiab])) OR "item discriminant"[tiab] OR "interscale correlation*"[tiab] OR error[tiab] OR errors[tiab] OR "individual variability"[tiab] OR "interval variability"[tiab] OR "rate variability"[tiab] OR (variability[tiab] AND (analysis[tiab] OR values[tiab])) OR (uncertainty[tiab] AND (measurement[tiab] OR measuring[tiab])) OR "standard error of measurement"[tiab] OR sensitiv*[tiab] OR responsive*[tiab] OR (limit[tiab] AND detection[tiab]) OR "minimal detectable concentration"[tiab] OR interpretab*[tiab] OR ((minimal[tiab] OR minimally[tiab] OR clinical[tiab] OR clinically[tiab]) AND (important[tiab] OR significant[tiab] OR detectable[tiab]) AND (change[tiab] OR difference[tiab])) OR (small*[tiab] AND (real[tiab] OR detectable[tiab]) AND (change[tiab] OR difference[tiab])) OR "meaningful change"[tiab] OR "ceiling effect"[tiab] OR "floor effect"[tiab] OR "Item response model"[tiab] OR IRT[tiab] OR Rasch[tiab] OR "Differential item functioning"[tiab] OR DIF[tiab] OR "computer adaptive testing"[tiab] OR "item bank"[tiab] OR "cross-cultural equivalence"[tiab]))) NOT (("addresses"[Publication Type] OR "biography"[Publication Type] OR "case reports"[Publication Type] OR "comment"[Publication Type] OR "directory"[Publication Type] OR "editorial"[Publication Type] OR "festschrift"[Publication Type] OR "interview"[Publication Type] OR "lectures"[Publication Type] OR "legal cases"[Publication Type] OR "legislation"[Publication Type] OR "letter"[Publication Type] OR "news"[Publication Type] OR "newspaper article"[Publication Type] OR "patient education handout"[Publication Type] OR "popular works"[Publication Type] OR "congresses"[Publication Type] OR "consensus development conference"[Publication Type] OR "consensus development conference, nih"[Publication Type] OR "practice guideline"[Publication Type]) NOT ("animals"[MeSH Terms] NOT "humans"[MeSH Terms]))

Wexner Cleveland Clinic Florida incontinence score
Hits: 98

Eligible: 0
((((Wexner Cleveland Clinic Florida incontinence score) OR (Wexner)) AND (anal fistula)) AND ((instrumentation[sh] OR methods[sh] OR "Validation Studies"[pt] OR "Comparative Study"[pt] OR "psychometrics"[MeSH] OR psychometr*[tiab] OR clinimetr*[tw] OR clinometr*[tw] OR "outcome assessment (health care)"[MeSH] OR "outcome assessment"[tiab] OR "outcome measure*"[tw] OR "observer variation"[MeSH] OR "observer variation"[tiab] OR "Health Status Indicators"[Mesh] OR "reproducibility of results"[MeSH] OR reproducib*[tiab] OR "discriminant analysis"[MeSH] OR reliab*[tiab] OR unreliab*[tiab] OR valid*[tiab] OR "coefficient of variation"[tiab] OR coefficient[tiab] OR homogeneity[tiab] OR homogeneous[tiab] OR "internal consistency"[tiab] OR (cronbach*[tiab] AND (alpha[tiab] OR alphas[tiab])) OR (item[tiab] AND (correlation*[tiab] OR selection*[tiab] OR reduction*[tiab])) OR agreement[tw] OR precision[tw] OR imprecision[tw] OR "precise values"[tw] OR test-retest[tiab] OR (test[tiab] AND retest[tiab]) OR (reliab*[tiab] AND (test[tiab] OR retest[tiab])) OR stability[tiab] OR interrater[tiab] OR inter-rater[tiab] OR intrarater[tiab] OR intra-rater[tiab] OR intertester[tiab] OR inter-tester[tiab] OR intratester[tiab] OR intra-tester[tiab] OR interobserver[tiab] OR inter-observer[tiab] OR intraobserver[tiab] OR intra-observer[tiab] OR intertechnician[tiab] OR inter-technician[tiab] OR intratechnician[tiab] OR intra-technician[tiab] OR interexaminer[tiab] OR inter-examiner[tiab] OR intraexaminer[tiab] OR intra-examiner[tiab] OR interassay[tiab] OR inter-assay[tiab] OR intraassay[tiab] OR intra-assay[tiab] OR interindividual[tiab] OR inter-individual[tiab] OR intraindividual[tiab] OR intra-individual[tiab] OR interparticipant[tiab] OR inter-participant[tiab] OR intraparticipant[tiab] OR intra-participant[tiab] OR kappa[tiab] OR kappa’s[tiab] OR kappas[tiab] OR repeatab*[tw] OR ((replicab*[tw] OR repeated[tw]) AND (measure[tw] OR measures[tw] OR findings[tw] OR result[tw] OR results[tw] OR test[tw] OR tests[tw])) OR generaliza*[tiab] OR generalisa*[tiab] OR concordance[tiab] OR (intraclass[tiab] AND correlation*[tiab]) OR discriminative[tiab] OR "known group"[tiab] OR "factor analysis"[tiab] OR "factor analyses"[tiab] OR "factor structure"[tiab] OR "factor structures"[tiab] OR dimension*[tiab] OR subscale*[tiab] OR (multitrait[tiab] AND scaling[tiab] AND (analysis[tiab] OR analyses[tiab])) OR "item discriminant"[tiab] OR "interscale correlation*"[tiab] OR error[tiab] OR errors[tiab] OR "individual variability"[tiab] OR "interval variability"[tiab] OR "rate variability"[tiab] OR (variability[tiab] AND (analysis[tiab] OR values[tiab])) OR (uncertainty[tiab] AND (measurement[tiab] OR measuring[tiab])) OR "standard error of measurement"[tiab] OR sensitiv*[tiab] OR responsive*[tiab] OR (limit[tiab] AND detection[tiab]) OR "minimal detectable concentration"[tiab] OR interpretab*[tiab] OR ((minimal[tiab] OR minimally[tiab] OR clinical[tiab] OR clinically[tiab]) AND (important[tiab] OR significant[tiab] OR detectable[tiab]) AND (change[tiab] OR difference[tiab])) OR (small*[tiab] AND (real[tiab] OR detectable[tiab]) AND (change[tiab] OR difference[tiab])) OR "meaningful change"[tiab] OR "ceiling effect"[tiab] OR "floor effect"[tiab] OR "Item response model"[tiab] OR IRT[tiab] OR Rasch[tiab] OR "Differential item functioning"[tiab] OR DIF[tiab] OR "computer adaptive testing"[tiab] OR "item bank"[tiab] OR "cross-cultural equivalence"[tiab]))) NOT (("addresses"[Publication Type] OR "biography"[Publication Type] OR "case reports"[Publication Type] OR "comment"[Publication Type] OR "directory"[Publication Type] OR "editorial"[Publication Type] OR "festschrift"[Publication Type] OR "interview"[Publication Type] OR "lectures"[Publication Type] OR "legal cases"[Publication Type] OR "legislation"[Publication Type] OR "letter"[Publication Type] OR "news"[Publication Type] OR "newspaper article"[Publication Type] OR "patient education handout"[Publication Type] OR "popular works"[Publication Type] OR "congresses"[Publication Type] OR "consensus development conference"[Publication Type] OR "consensus development conference, nih"[Publication Type] OR "practice guideline"[Publication Type]) NOT ("animals"[MeSH Terms] NOT "humans"[MeSH Terms]))

Vaizey incontinence score
Hits: 14
Eligible: 0
((((Vaizey incontinence score) OR (Vaizey)) AND (anal fistula)) AND ((instrumentation[sh] OR methods[sh] OR "Validation Studies"[pt] OR "Comparative Study"[pt] OR "psychometrics"[MeSH] OR psychometr*[tiab] OR clinimetr*[tw] OR clinometr*[tw] OR "outcome assessment (health care)"[MeSH] OR "outcome assessment"[tiab] OR "outcome measure*"[tw] OR "observer variation"[MeSH] OR "observer variation"[tiab] OR "Health Status Indicators"[Mesh] OR "reproducibility of results"[MeSH] OR reproducib*[tiab] OR "discriminant analysis"[MeSH] OR reliab*[tiab] OR unreliab*[tiab] OR valid*[tiab] OR "coefficient of variation"[tiab] OR coefficient[tiab] OR homogeneity[tiab] OR homogeneous[tiab] OR "internal consistency"[tiab] OR (cronbach*[tiab] AND (alpha[tiab] OR alphas[tiab])) OR (item[tiab] AND (correlation*[tiab] OR selection*[tiab] OR reduction*[tiab])) OR agreement[tw] OR precision[tw] OR imprecision[tw] OR "precise values"[tw] OR test-retest[tiab] OR (test[tiab] AND retest[tiab]) OR (reliab*[tiab] AND (test[tiab] OR retest[tiab])) OR stability[tiab] OR interrater[tiab] OR inter-rater[tiab] OR intrarater[tiab] OR intra-rater[tiab] OR intertester[tiab] OR inter-tester[tiab] OR intratester[tiab] OR intra-tester[tiab]

OR interobserver[tiab] OR inter-observer[tiab] OR intraobserver[tiab] OR intra-observer[tiab] OR intertechnician[tiab] OR inter-technician[tiab] OR intratechnician[tiab] OR intra-technician[tiab] OR interexaminer[tiab] OR inter-examiner[tiab] OR intraexaminer[tiab] OR intra-examiner[tiab] OR interassay[tiab] OR inter-assay[tiab] OR intraassay[tiab] OR intra-assay[tiab] OR interindividual[tiab] OR inter-individual[tiab] OR intraindividual[tiab] OR intra-individual[tiab] OR interparticipant[tiab] OR inter-participant[tiab] OR intraparticipant[tiab] OR intra-participant[tiab] OR kappa[tiab] OR kappa's[tiab] OR kappas[tiab] OR repeatab*[tw] OR ((replicab*[tw] OR repeated[tw]) AND (measure[tw] OR measures[tw] OR findings[tw] OR result[tw] OR results[tw] OR test[tw] OR tests[tw])) OR generaliza*[tiab] OR generalisa*[tiab] OR concordance[tiab] OR (intraclass[tiab] AND correlation*[tiab]) OR discriminative[tiab] OR "known group"[tiab] OR "factor analysis"[tiab] OR "factor analyses"[tiab] OR "factor structure"[tiab] OR "factor structures"[tiab] OR dimension*[tiab] OR subscale*[tiab] OR (multitrait[tiab] AND scaling[tiab] AND (analysis[tiab] OR analyses[tiab])) OR "item discriminant"[tiab] OR "interscale correlation*"[tiab] OR error[tiab] OR errors[tiab] OR "individual variability"[tiab] OR "interval variability"[tiab] OR "rate variability"[tiab] OR (variability[tiab] AND (analysis[tiab] OR values[tiab])) OR (uncertainty[tiab] AND (measurement[tiab] OR measuring[tiab])) OR "standard error of measurement"[tiab] OR sensitiv*[tiab] OR responsive*[tiab] OR (limit[tiab] AND detection[tiab]) OR "minimal detectable concentration"[tiab] OR interpretab*[tiab] OR ((minimal[tiab] OR minimally[tiab] OR clinical[tiab] OR clinically[tiab]) AND (important[tiab] OR significant[tiab] OR detectable[tiab]) AND (change[tiab] OR difference[tiab])) OR (small*[tiab] AND (real[tiab] OR detectable[tiab]) AND (change[tiab] OR difference[tiab])) OR "meaningful change"[tiab] OR "ceiling effect"[tiab] OR "floor effect"[tiab] OR "Item response model"[tiab] OR IRT[tiab] OR Rasch[tiab] OR "Differential item functioning"[tiab] OR DIF[tiab] OR "computer adaptive testing"[tiab] OR "item bank"[tiab] OR "cross-cultural equivalence"[tiab]))) NOT (("addresses"[Publication Type] OR "biography"[Publication Type] OR "case reports"[Publication Type] OR "comment"[Publication Type] OR "directory"[Publication Type] OR "editorial"[Publication Type] OR "festschrift"[Publication Type] OR "interview"[Publication Type] OR "lectures"[Publication Type] OR "legal cases"[Publication Type] OR "legislation"[Publication Type] OR "letter"[Publication Type] OR "news"[Publication Type] OR "newspaper article"[Publication Type] OR "patient education handout"[Publication Type] OR "popular works"[Publication Type] OR "congresses"[Publication Type] OR "consensus development conference"[Publication Type] OR "consensus development conference, nih"[Publication Type] OR "practice guideline"[Publication Type]) NOT ("animals"[MeSH Terms] NOT "humans"[MeSH Terms]))

Fecal Incontinence Quality of Life Scale
Hits: 23
Eligible: 0
((((Fecal Incontinence Quality of Life Scale) OR (FIQL)) AND (anal fistula)) AND ((instrumentation[sh] OR methods[sh] OR "Validation Studies"[pt] OR "Comparative Study"[pt] OR "psychometrics"[MeSH] OR psychometr*[tiab] OR clinimetr*[tw] OR clinometr*[tw] OR "outcome assessment (health care)"[MeSH] OR "outcome assessment"[tiab] OR "outcome measure*"[tw] OR "observer variation"[MeSH] OR "observer variation"[tiab] OR "Health Status Indicators"[Mesh] OR "reproducibility of results"[MeSH] OR reproducib*[tiab] OR "discriminant analysis"[MeSH] OR reliab*[tiab] OR unreliab*[tiab] OR valid*[tiab] OR "coefficient of variation"[tiab] OR coefficient[tiab] OR homogeneity[tiab] OR homogeneous[tiab] OR "internal consistency"[tiab] OR (cronbach*[tiab] AND (alpha[tiab] OR alphas[tiab])) OR (item[tiab] AND (correlation*[tiab] OR selection*[tiab] OR reduction*[tiab])) OR agreement[tw] OR precision[tw] OR imprecision[tw] OR "precise values"[tw] OR test-retest[tiab] OR (test[tiab] AND retest[tiab]) OR (reliab*[tiab] AND (test[tiab] OR retest[tiab])) OR stability[tiab] OR interrater[tiab] OR inter-rater[tiab] OR intrarater[tiab] OR intra-rater[tiab] OR intertester[tiab] OR inter-tester[tiab] OR intratester[tiab] OR intra-tester[tiab] OR interobserver[tiab] OR inter-observer[tiab] OR intraobserver[tiab] OR intra-observer[tiab] OR intertechnician[tiab] OR inter-technician[tiab] OR intratechnician[tiab] OR intra-technician[tiab] OR interexaminer[tiab] OR inter-examiner[tiab] OR intraexaminer[tiab] OR intra-examiner[tiab] OR interassay[tiab] OR inter-assay[tiab] OR intraassay[tiab] OR intra-assay[tiab] OR interindividual[tiab] OR inter-individual[tiab] OR intraindividual[tiab] OR intra-individual[tiab] OR interparticipant[tiab] OR inter-participant[tiab] OR intraparticipant[tiab] OR intra-participant[tiab] OR kappa[tiab] OR kappa's[tiab] OR kappas[tiab] OR repeatab*[tw] OR ((replicab*[tw] OR repeated[tw]) AND (measure[tw] OR measures[tw] OR findings[tw] OR result[tw] OR results[tw] OR test[tw] OR tests[tw])) OR generaliza*[tiab] OR generalisa*[tiab] OR concordance[tiab] OR (intraclass[tiab] AND correlation*[tiab]) OR discriminative[tiab] OR "known group"[tiab] OR "factor analysis"[tiab] OR "factor analyses"[tiab] OR "factor structure"[tiab] OR "factor structures"[tiab] OR dimension*[tiab] OR subscale*[tiab] OR (multitrait[tiab] AND scaling[tiab] AND (analysis[tiab] OR analyses[tiab])) OR "item discriminant"[tiab] OR "interscale correlation*"[tiab] OR error[tiab] OR errors[tiab] OR "individual

variability"[tiab] OR "interval variability"[tiab] OR "rate variability"[tiab] OR (variability[tiab] AND (analysis[tiab] OR values[tiab])) OR (uncertainty[tiab] AND (measurement[tiab] OR measuring[tiab])) OR "standard error of measurement"[tiab] OR sensitiv*[tiab] OR responsive*[tiab] OR (limit[tiab] AND detection[tiab]) OR "minimal detectable concentration"[tiab] OR interpretab*[tiab] OR ((minimal[tiab] OR minimally[tiab] OR clinical[tiab] OR clinically[tiab]) AND (important[tiab] OR significant[tiab] OR detectable[tiab]) AND (change[tiab] OR difference[tiab])) OR (small*[tiab] AND (real[tiab] OR detectable[tiab]) AND (change[tiab] OR difference[tiab])) OR "meaningful change"[tiab] OR "ceiling effect"[tiab] OR "floor effect"[tiab] OR "Item response model"[tiab] OR IRT[tiab] OR Rasch[tiab] OR "Differential item functioning"[tiab] OR DIF[tiab] OR "computer adaptive testing"[tiab] OR "item bank"[tiab] OR "cross-cultural equivalence"[tiab]))) NOT (("addresses"[Publication Type] OR "biography"[Publication Type] OR "case reports"[Publication Type] OR "comment"[Publication Type] OR "directory"[Publication Type] OR "editorial"[Publication Type] OR "festschrift"[Publication Type] OR "interview"[Publication Type] OR "lectures"[Publication Type] OR "legal cases"[Publication Type] OR "legislation"[Publication Type] OR "letter"[Publication Type] OR "news"[Publication Type] OR "newspaper article"[Publication Type] OR "patient education handout"[Publication Type] OR "popular works"[Publication Type] OR "congresses"[Publication Type] OR "consensus development conference"[Publication Type] OR "consensus development conference, nih"[Publication Type] OR "practice guideline"[Publication Type]) NOT ("animals"[MeSH Terms] NOT "humans"[MeSH Terms]))

Colorectal functional outcome questionnaire
Hits: 29
Eligible: 0
(((Colorectal functional outcome questionnaire) AND (anal fistula)) AND ((instrumentation[sh] OR methods[sh] OR "Validation Studies"[pt] OR "Comparative Study"[pt] OR "psychometrics"[MeSH] OR psychometr*[tiab] OR clinimetr*[tw] OR clinometr*[tw] OR "outcome assessment (health care)"[MeSH] OR "outcome assessment"[tiab] OR "outcome measure*"[tw] OR "observer variation"[MeSH] OR "observer variation"[tiab] OR "Health Status Indicators"[Mesh] OR "reproducibility of results"[MeSH] OR reproducib*[tiab] OR "discriminant analysis"[MeSH] OR reliab*[tiab] OR unreliab*[tiab] OR valid*[tiab] OR "coefficient of variation"[tiab] OR coefficient[tiab] OR homogeneity[tiab] OR homogeneous[tiab] OR "internal consistency"[tiab] OR (cronbach*[tiab] AND (alpha[tiab] OR alphas[tiab])) OR (item[tiab] AND (correlation*[tiab] OR selection*[tiab] OR reduction*[tiab])) OR agreement[tw] OR precision[tw] OR imprecision[tw] OR "precise values"[tw] OR test-retest[tiab] OR (test[tiab] AND retest[tiab]) OR (reliab*[tiab] AND (test[tiab] OR retest[tiab])) OR stability[tiab] OR interrater[tiab] OR inter-rater[tiab] OR intrarater[tiab] OR intra-rater[tiab] OR intertester[tiab] OR inter-tester[tiab] OR intratester[tiab] OR intra-tester[tiab] OR interobserver[tiab] OR inter-observer[tiab] OR intraobserver[tiab] OR intra-observer[tiab] OR intertechnician[tiab] OR inter-technician[tiab] OR intratechnician[tiab] OR intra-technician[tiab] OR interexaminer[tiab] OR inter-examiner[tiab] OR intraexaminer[tiab] OR intra-examiner[tiab] OR interassay[tiab] OR inter-assay[tiab] OR intraassay[tiab] OR intra-assay[tiab] OR interindividual[tiab] OR inter-individual[tiab] OR intraindividual[tiab] OR intra-individual[tiab] OR interparticipant[tiab] OR inter-participant[tiab] OR intraparticipant[tiab] OR intra-participant[tiab] OR kappa[tiab] OR kappa's[tiab] OR kappas[tiab] OR repeatab*[tw] OR ((replicab*[tw] OR repeated[tw]) AND (measure[tw] OR measures[tw] OR findings[tw] OR result[tw] OR results[tw] OR test[tw] OR tests[tw])) OR generaliza*[tiab] OR generalisa*[tiab] OR concordance[tiab] OR (intraclass[tiab] AND correlation*[tiab]) OR discriminative[tiab] OR "known group"[tiab] OR "factor analysis"[tiab] OR "factor analyses"[tiab] OR "factor structure"[tiab] OR "factor structures"[tiab] OR dimension*[tiab] OR subscale*[tiab] OR (multitrait[tiab] AND scaling[tiab] AND (analysis[tiab] OR analyses[tiab])) OR "item discriminant"[tiab] OR "interscale correlation*"[tiab] OR error[tiab] OR errors[tiab] OR "individual variability"[tiab] OR "interval variability"[tiab] OR "rate variability"[tiab] OR (variability[tiab] AND (analysis[tiab] OR values[tiab])) OR (uncertainty[tiab] AND (measurement[tiab] OR measuring[tiab])) OR "standard error of measurement"[tiab] OR sensitiv*[tiab] OR responsive*[tiab] OR (limit[tiab] AND detection[tiab]) OR "minimal detectable concentration"[tiab] OR interpretab*[tiab] OR ((minimal[tiab] OR minimally[tiab] OR clinical[tiab] OR clinically[tiab]) AND (important[tiab] OR significant[tiab] OR detectable[tiab]) AND (change[tiab] OR difference[tiab])) OR (small*[tiab] AND (real[tiab] OR detectable[tiab]) AND (change[tiab] OR difference[tiab])) OR "meaningful change"[tiab] OR "ceiling effect"[tiab] OR "floor effect"[tiab] OR "Item response model"[tiab] OR IRT[tiab] OR Rasch[tiab] OR "Differential item functioning"[tiab] OR DIF[tiab] OR "computer adaptive testing"[tiab] OR "item bank"[tiab] OR "cross-cultural equivalence"[tiab]))) NOT (("addresses"[Publication Type] OR "biography"[Publication Type] OR "case reports"[Publication Type] OR "comment"[Publication Type] OR "directory"[Publication Type] OR "editorial"[Publication Type] OR "festschrift"[Publication Type] OR "interview"[Publication Type] OR "lectures"[Publication Type] OR "legal cases"[Publication Type] OR

"legislation"[Publication Type] OR "letter"[Publication Type] OR "news"[Publication Type] OR "newspaper article"[Publication Type] OR "patient education handout"[Publication Type] OR "popular works"[Publication Type] OR "congresses"[Publication Type] OR "consensus development conference"[Publication Type] OR "consensus development conference, nih"[Publication Type] OR "practice guideline"[Publication Type]) NOT ("animals"[MeSH Terms] NOT "humans"[MeSH Terms]))

St. Mark's Incontinence Score (SMIS)
Hits: 10
Eligible: 0
((((St. Mark's Incontinence Score) OR (SMIS)) AND (anal fistula)) AND ((instrumentation[sh] OR methods[sh] OR "Validation Studies"[pt] OR "Comparative Study"[pt] OR "psychometrics"[MeSH] OR psychometr*[tiab] OR clinimetr*[tw] OR clinometr*[tw] OR "outcome assessment (health care)"[MeSH] OR "outcome assessment"[tiab] OR "outcome measure*"[tw] OR "observer variation"[MeSH] OR "observer variation"[tiab] OR "Health Status Indicators"[Mesh] OR "reproducibility of results"[MeSH] OR reproducib*[tiab] OR "discriminant analysis"[MeSH] OR reliab*[tiab] OR unreliab*[tiab] OR valid*[tiab] OR "coefficient of variation"[tiab] OR coefficient[tiab] OR homogeneity[tiab] OR homogeneous[tiab] OR "internal consistency"[tiab] OR (cronbach*[tiab] AND (alpha[tiab] OR alphas[tiab])) OR (item[tiab] AND (correlation*[tiab] OR selection*[tiab] OR reduction*[tiab])) OR agreement[tw] OR precision[tw] OR imprecision[tw] OR "precise values"[tw] OR test-retest[tiab] OR (test[tiab] AND retest[tiab]) OR (reliab*[tiab] AND (test[tiab] OR retest[tiab])) OR stability[tiab] OR interrater[tiab] OR inter-rater[tiab] OR intrarater[tiab] OR intra-rater[tiab] OR intertester[tiab] OR inter-tester[tiab] OR intratester[tiab] OR intra-tester[tiab] OR interobserver[tiab] OR inter-observer[tiab] OR intraobserver[tiab] OR intra-observer[tiab] OR intertechnician[tiab] OR inter-technician[tiab] OR intratechnician[tiab] OR intra-technician[tiab] OR interexaminer[tiab] OR inter-examiner[tiab] OR intraexaminer[tiab] OR intra-examiner[tiab] OR interassay[tiab] OR inter-assay[tiab] OR intraassay[tiab] OR intra-assay[tiab] OR interindividual[tiab] OR inter-individual[tiab] OR intraindividual[tiab] OR intra-individual[tiab] OR interparticipant[tiab] OR inter-participant[tiab] OR intraparticipant[tiab] OR intra-participant[tiab] OR kappa[tiab] OR kappa’s[tiab] OR kappas[tiab] OR repeatab*[tw] OR ((replicab*[tw] OR repeated[tw]) AND (measure[tw] OR measures[tw] OR findings[tw] OR result[tw] OR results[tw] OR test[tw] OR tests[tw])) OR generaliza*[tiab] OR generalisa*[tiab] OR concordance[tiab] OR (intraclass[tiab] AND correlation*[tiab]) OR discriminative[tiab] OR "known group"[tiab] OR "factor analysis"[tiab] OR "factor analyses"[tiab] OR "factor structure"[tiab] OR "factor structures"[tiab] OR dimension*[tiab] OR subscale*[tiab] OR (multitrait[tiab] AND scaling[tiab] AND (analysis[tiab] OR analyses[tiab])) OR "item discriminant"[tiab] OR "interscale correlation*"[tiab] OR error[tiab] OR errors[tiab] OR "individual variability"[tiab] OR "interval variability"[tiab] OR "rate variability"[tiab] OR (variability[tiab] AND (analysis[tiab] OR values[tiab])) OR (uncertainty[tiab] AND (measurement[tiab] OR measuring[tiab])) OR "standard error of measurement"[tiab] OR sensitiv*[tiab] OR responsive*[tiab] OR (limit[tiab] AND detection[tiab]) OR "minimal detectable concentration"[tiab] OR interpretab*[tiab] OR ((minimal[tiab] OR minimally[tiab] OR clinical[tiab] OR clinically[tiab]) AND (important[tiab] OR significant[tiab] OR detectable[tiab]) AND (change[tiab] OR difference[tiab])) OR (small*[tiab] AND (real[tiab] OR detectable[tiab]) AND (change[tiab] OR difference[tiab])) OR "meaningful change"[tiab] OR "ceiling effect"[tiab] OR "floor effect"[tiab] OR "Item response model"[tiab] OR IRT[tiab] OR Rasch[tiab] OR "Differential item functioning"[tiab] OR DIF[tiab] OR "computer adaptive testing"[tiab] OR "item bank"[tiab] OR "cross-cultural equivalence"[tiab]))) NOT (("addresses"[Publication Type] OR "biography"[Publication Type] OR "case reports"[Publication Type] OR "comment"[Publication Type] OR "directory"[Publication Type] OR "editorial"[Publication Type] OR "festschrift"[Publication Type] OR "interview"[Publication Type] OR "lectures"[Publication Type] OR "legal cases"[Publication Type] OR "legislation"[Publication Type] OR "letter"[Publication Type] OR "news"[Publication Type] OR "newspaper article"[Publication Type] OR "patient education handout"[Publication Type] OR "popular works"[Publication Type] OR "congresses"[Publication Type] OR "consensus development conference"[Publication Type] OR "consensus development conference, nih"[Publication Type] OR "practice guideline"[Publication Type]) NOT ("animals"[MeSH Terms] NOT "humans"[MeSH Terms]))

German Society of Coloproctology Score
Hits: 1
Eligible: 0
(((German Society of Coloproctology score) AND (anal fistula)) AND ((instrumentation[sh] OR methods[sh] OR "Validation Studies"[pt] OR "Comparative Study"[pt] OR "psychometrics"[MeSH] OR psychometr*[tiab] OR clinimetr*[tw] OR clinometr*[tw] OR "outcome assessment (health care)"[MeSH] OR "outcome assessment"[tiab]

OR "outcome measure*"[tw] OR "observer variation"[MeSH] OR "observer variation"[tiab] OR "Health Status Indicators"[Mesh] OR "reproducibility of results"[MeSH] OR reproducib*[tiab] OR "discriminant analysis"[MeSH] OR reliab*[tiab] OR unreliab*[tiab] OR valid*[tiab] OR "coefficient of variation"[tiab] OR coefficient[tiab] OR homogeneity[tiab] OR homogeneous[tiab] OR "internal consistency"[tiab] OR (cronbach*[tiab] AND (alpha[tiab] OR alphas[tiab])) OR (item[tiab] AND (correlation*[tiab] OR selection*[tiab] OR reduction*[tiab])) OR agreement[tw] OR precision[tw] OR imprecision[tw] OR "precise values"[tw] OR test-retest[tiab] OR (test[tiab] AND retest[tiab]) OR (reliab*[tiab] AND (test[tiab] OR retest[tiab])) OR stability[tiab] OR interrater[tiab] OR inter-rater[tiab] OR intrarater[tiab] OR intra-rater[tiab] OR intertester[tiab] OR inter-tester[tiab] OR intratester[tiab] OR intra-tester[tiab] OR interobserver[tiab] OR inter-observer[tiab] OR intraobserver[tiab] OR intra-observer[tiab] OR intertechnician[tiab] OR inter-technician[tiab] OR intratechnician[tiab] OR intra-technician[tiab] OR interexaminer[tiab] OR inter-examiner[tiab] OR intraexaminer[tiab] OR intra-examiner[tiab] OR interassay[tiab] OR inter-assay[tiab] OR intraassay[tiab] OR intra-assay[tiab] OR interindividual[tiab] OR inter-individual[tiab] OR intraindividual[tiab] OR intra-individual[tiab] OR interparticipant[tiab] OR inter-participant[tiab] OR intraparticipant[tiab] OR intra-participant[tiab] OR kappa[tiab] OR kappa's[tiab] OR kappas[tiab] OR repeatab*[tw] OR ((replicab*[tw] OR repeated[tw]) AND (measure[tw] OR measures[tw] OR findings[tw] OR result[tw] OR results[tw] OR test[tw] OR tests[tw])) OR generaliza*[tiab] OR generalisa*[tiab] OR concordance[tiab] OR (intraclass[tiab] AND correlation*[tiab]) OR discriminative[tiab] OR "known group"[tiab] OR "factor analysis"[tiab] OR "factor analyses"[tiab] OR "factor structure"[tiab] OR "factor structures"[tiab] OR dimension*[tiab] OR subscale*[tiab] OR (multitrait[tiab] AND scaling[tiab] AND (analysis[tiab] OR analyses[tiab])) OR "item discriminant"[tiab] OR "interscale correlation*"[tiab] OR error[tiab] OR errors[tiab] OR "individual variability"[tiab] OR "interval variability"[tiab] OR "rate variability"[tiab] OR (variability[tiab] AND (analysis[tiab] OR values[tiab])) OR (uncertainty[tiab] AND (measurement[tiab] OR measuring[tiab])) OR "standard error of measurement"[tiab] OR sensitiv*[tiab] OR responsive*[tiab] OR (limit[tiab] AND detection[tiab]) OR "minimal detectable concentration"[tiab] OR interpretab*[tiab] OR ((minimal[tiab] OR minimally[tiab] OR clinical[tiab] OR clinically[tiab]) AND (important[tiab] OR significant[tiab] OR detectable[tiab]) AND (change[tiab] OR difference[tiab])) OR (small*[tiab] AND (real[tiab] OR detectable[tiab]) AND (change[tiab] OR difference[tiab])) OR "meaningful change"[tiab] OR "ceiling effect"[tiab] OR "floor effect"[tiab] OR "Item response model"[tiab] OR IRT[tiab] OR Rasch[tiab] OR "Differential item functioning"[tiab] OR DIF[tiab] OR "computer adaptive testing"[tiab] OR "item bank"[tiab] OR "cross-cultural equivalence"[tiab]))) NOT (("addresses"[Publication Type] OR "biography"[Publication Type] OR "case reports"[Publication Type] OR "comment"[Publication Type] OR "directory"[Publication Type] OR "editorial"[Publication Type] OR "festschrift"[Publication Type] OR "interview"[Publication Type] OR "lectures"[Publication Type] OR "legal cases"[Publication Type] OR "legislation"[Publication Type] OR "letter"[Publication Type] OR "news"[Publication Type] OR "newspaper article"[Publication Type] OR "patient education handout"[Publication Type] OR "popular works"[Publication Type] OR "congresses"[Publication Type] OR "consensus development conference"[Publication Type] OR "consensus development conference, nih"[Publication Type] OR "practice guideline"[Publication Type]) NOT ("animals"[MeSH Terms] NOT "humans"[MeSH Terms]))

Williams grade
Hits: 3
Eligible: 0
(((Williams grade) AND (anal fistula)) AND ((instrumentation[sh] OR methods[sh] OR "Validation Studies"[pt] OR "Comparative Study"[pt] OR "psychometrics"[MeSH] OR psychometr*[tiab] OR clinimetr*[tw] OR clinometr*[tw] OR "outcome assessment (health care)"[MeSH] OR "outcome assessment"[tiab] OR "outcome measure*"[tw] OR "observer variation"[MeSH] OR "observer variation"[tiab] OR "Health Status Indicators"[Mesh] OR "reproducibility of results"[MeSH] OR reproducib*[tiab] OR "discriminant analysis"[MeSH] OR reliab*[tiab] OR unreliab*[tiab] OR valid*[tiab] OR "coefficient of variation"[tiab] OR coefficient[tiab] OR homogeneity[tiab] OR homogeneous[tiab] OR "internal consistency"[tiab] OR (cronbach*[tiab] AND (alpha[tiab] OR alphas[tiab])) OR (item[tiab] AND (correlation*[tiab] OR selection*[tiab] OR reduction*[tiab])) OR agreement[tw] OR precision[tw] OR imprecision[tw] OR "precise values"[tw] OR test-retest[tiab] OR (test[tiab] AND retest[tiab]) OR (reliab*[tiab] AND (test[tiab] OR retest[tiab])) OR stability[tiab] OR interrater[tiab] OR inter-rater[tiab] OR intrarater[tiab] OR intra-rater[tiab] OR intertester[tiab] OR inter-tester[tiab] OR intratester[tiab] OR intra-tester[tiab] OR interobserver[tiab] OR inter-observer[tiab] OR intraobserver[tiab] OR intra-observer[tiab] OR intertechnician[tiab] OR inter-technician[tiab] OR intratechnician[tiab] OR intra-technician[tiab] OR interexaminer[tiab] OR inter-examiner[tiab] OR intraexaminer[tiab] OR intra-examiner[tiab] OR interassay[tiab] OR inter-assay[tiab] OR intraassay[tiab] OR intra-assay[tiab] OR interindividual[tiab] OR inter-individual[tiab] OR intraindividual[tiab] OR intra-individual[tiab]

OR interparticipant[tiab] OR inter-participant[tiab] OR intraparticipant[tiab] OR intra-participant[tiab] OR kappa[tiab] OR kappa's[tiab] OR kappas[tiab] OR repeatab*[tw] OR ((replicab*[tw] OR repeated[tw]) AND (measure[tw] OR measures[tw] OR findings[tw] OR result[tw] OR results[tw] OR test[tw] OR tests[tw])) OR generaliza*[tiab] OR generalisa*[tiab] OR concordance[tiab] OR (intraclass[tiab] AND correlation*[tiab]) OR discriminative[tiab] OR "known group"[tiab] OR "factor analysis"[tiab] OR "factor analyses"[tiab] OR "factor structure"[tiab] OR "factor structures"[tiab] OR dimension*[tiab] OR subscale*[tiab] OR (multitrait[tiab] AND scaling[tiab] AND (analysis[tiab] OR analyses[tiab])) OR "item discriminant"[tiab] OR "interscale correlation*"[tiab] OR error[tiab] OR errors[tiab] OR "individual variability"[tiab] OR "interval variability"[tiab] OR "rate variability"[tiab] OR (variability[tiab] AND (analysis[tiab] OR values[tiab])) OR (uncertainty[tiab] AND (measurement[tiab] OR measuring[tiab])) OR "standard error of measurement"[tiab] OR sensitiv*[tiab] OR responsive*[tiab] OR (limit[tiab] AND detection[tiab]) OR "minimal detectable concentration"[tiab] OR interpretab*[tiab] OR ((minimal[tiab] OR minimally[tiab] OR clinical[tiab] OR clinically[tiab]) AND (important[tiab] OR significant[tiab] OR detectable[tiab]) AND (change[tiab] OR difference[tiab])) OR (small*[tiab] AND (real[tiab] OR detectable[tiab]) AND (change[tiab] OR difference[tiab])) OR "meaningful change"[tiab] OR "ceiling effect"[tiab] OR "floor effect"[tiab] OR "Item response model"[tiab] OR IRT[tiab] OR Rasch[tiab] OR "Differential item functioning"[tiab] OR DIF[tiab] OR "computer adaptive testing"[tiab] OR "item bank"[tiab] OR "cross-cultural equivalence"[tiab]))) NOT (("addresses"[Publication Type] OR "biography"[Publication Type] OR "case reports"[Publication Type] OR "comment"[Publication Type] OR "directory"[Publication Type] OR "editorial"[Publication Type] OR "festschrift"[Publication Type] OR "interview"[Publication Type] OR "lectures"[Publication Type] OR "legal cases"[Publication Type] OR "legislation"[Publication Type] OR "letter"[Publication Type] OR "news"[Publication Type] OR "newspaper article"[Publication Type] OR "patient education handout"[Publication Type] OR "popular works"[Publication Type] OR "congresses"[Publication Type] OR "consensus development conference"[Publication Type] OR "consensus development conference, nih"[Publication Type] OR "practice guideline"[Publication Type]) NOT ("animals"[MeSH Terms] NOT "humans"[MeSH Terms]))

Short Form-36 health survey (SF-36)
Hits: 14
Eligible: 0

((((Short Form-36 health Survey) OR (SF-36)) AND (anal fistula)) AND ((instrumentation[sh] OR methods[sh] OR "Validation Studies"[pt] OR "Comparative Study"[pt] OR "psychometrics"[MeSH] OR psychometr*[tiab] OR clinimetr*[tw] OR clinometr*[tw] OR "outcome assessment (health care)"[MeSH] OR "outcome assessment"[tiab] OR "outcome measure*"[tw] OR "observer variation"[MeSH] OR "observer variation"[tiab] OR "Health Status Indicators"[Mesh] OR "reproducibility of results"[MeSH] OR reproducib*[tiab] OR "discriminant analysis"[MeSH] OR reliab*[tiab] OR unreliab*[tiab] OR valid*[tiab] OR "coefficient of variation"[tiab] OR coefficient[tiab] OR homogeneity[tiab] OR homogeneous[tiab] OR "internal consistency"[tiab] OR (cronbach*[tiab] AND (alpha[tiab] OR alphas[tiab])) OR (item[tiab] AND (correlation*[tiab] OR selection*[tiab] OR reduction*[tiab])) OR agreement[tw] OR precision[tw] OR imprecision[tw] OR "precise values"[tw] OR test-retest[tiab] OR (test[tiab] AND retest[tiab]) OR (reliab*[tiab] AND (test[tiab] OR retest[tiab])) OR stability[tiab] OR interrater[tiab] OR inter-rater[tiab] OR intrarater[tiab] OR intra-rater[tiab] OR intertester[tiab] OR inter-tester[tiab] OR intratester[tiab] OR intra-tester[tiab] OR interobserver[tiab] OR inter-observer[tiab] OR intraobserver[tiab] OR intra-observer[tiab] OR intertechnician[tiab] OR inter-technician[tiab] OR intratechnician[tiab] OR intra-technician[tiab] OR interexaminer[tiab] OR inter-examiner[tiab] OR intraexaminer[tiab] OR intra-examiner[tiab] OR interassay[tiab] OR inter-assay[tiab] OR intraassay[tiab] OR intra-assay[tiab] OR interindividual[tiab] OR inter-individual[tiab] OR intraindividual[tiab] OR intra-individual[tiab] OR interparticipant[tiab] OR inter-participant[tiab] OR intraparticipant[tiab] OR intra-participant[tiab] OR kappa[tiab] OR kappa's[tiab] OR kappas[tiab] OR repeatab*[tw] OR ((replicab*[tw] OR repeated[tw]) AND (measure[tw] OR measures[tw] OR findings[tw] OR result[tw] OR results[tw] OR test[tw] OR tests[tw])) OR generaliza*[tiab] OR generalisa*[tiab] OR concordance[tiab] OR (intraclass[tiab] AND correlation*[tiab]) OR discriminative[tiab] OR "known group"[tiab] OR "factor analysis"[tiab] OR "factor analyses"[tiab] OR "factor structure"[tiab] OR "factor structures"[tiab] OR dimension*[tiab] OR subscale*[tiab] OR (multitrait[tiab] AND scaling[tiab] AND (analysis[tiab] OR analyses[tiab])) OR "item discriminant"[tiab] OR "interscale correlation*"[tiab] OR error[tiab] OR errors[tiab] OR "individual variability"[tiab] OR "interval variability"[tiab] OR "rate variability"[tiab] OR (variability[tiab] AND (analysis[tiab] OR values[tiab])) OR (uncertainty[tiab] AND (measurement[tiab] OR measuring[tiab])) OR "standard error of measurement"[tiab] OR sensitiv*[tiab] OR responsive*[tiab] OR (limit[tiab] AND detection[tiab]) OR "minimal detectable concentration"[tiab] OR interpretab*[tiab] OR ((minimal[tiab] OR minimally[tiab] OR clinical[tiab] OR clinically[tiab]) AND (important[tiab] OR significant[tiab] OR detectable[tiab]) AND (change[tiab] OR

difference[tiab])) OR (small*[tiab] AND (real[tiab] OR detectable[tiab]) AND (change[tiab] OR difference[tiab])) OR "meaningful change"[tiab] OR "ceiling effect"[tiab] OR "floor effect"[tiab] OR "Item response model"[tiab] OR IRT[tiab] OR Rasch[tiab] OR "Differential item functioning"[tiab] OR DIF[tiab] OR "computer adaptive testing"[tiab] OR "item bank"[tiab] OR "cross-cultural equivalence"[tiab]))) NOT (("addresses"[Publication Type] OR "biography"[Publication Type] OR "case reports"[Publication Type] OR "comment"[Publication Type] OR "directory"[Publication Type] OR "editorial"[Publication Type] OR "festschrift"[Publication Type] OR "interview"[Publication Type] OR "lectures"[Publication Type] OR "legal cases"[Publication Type] OR "legislation"[Publication Type] OR "letter"[Publication Type] OR "news"[Publication Type] OR "newspaper article"[Publication Type] OR "patient education handout"[Publication Type] OR "popular works"[Publication Type] OR "congresses"[Publication Type] OR "consensus development conference"[Publication Type] OR "consensus development conference, nih"[Publication Type] OR "practice guideline"[Publication Type]) NOT ("animals"[MeSH Terms] NOT "humans"[MeSH Terms]))

EQ-5D
Hits: 3
Eligible: 0
(((EQ-5D) AND (anal fistula)) AND ((instrumentation[sh] OR methods[sh] OR "Validation Studies"[pt] OR "Comparative Study"[pt] OR "psychometrics"[MeSH] OR psychometr*[tiab] OR clinimetr*[tw] OR clinometr*[tw] OR "outcome assessment (health care)"[MeSH] OR "outcome assessment"[tiab] OR "outcome measure*"[tw] OR "observer variation"[MeSH] OR "observer variation"[tiab] OR "Health Status Indicators"[Mesh] OR "reproducibility of results"[MeSH] OR reproducib*[tiab] OR "discriminant analysis"[MeSH] OR reliab*[tiab] OR unreliab*[tiab] OR valid*[tiab] OR "coefficient of variation"[tiab] OR coefficient[tiab] OR homogeneity[tiab] OR homogeneous[tiab] OR "internal consistency"[tiab] OR (cronbach*[tiab] AND (alpha[tiab] OR alphas[tiab])) OR (item[tiab] AND (correlation*[tiab] OR selection*[tiab] OR reduction*[tiab])) OR agreement[tw] OR precision[tw] OR imprecision[tw] OR "precise values"[tw] OR test-retest[tiab] OR (test[tiab] AND retest[tiab]) OR (reliab*[tiab] AND (test[tiab] OR retest[tiab])) OR stability[tiab] OR interrater[tiab] OR inter-rater[tiab] OR intrarater[tiab] OR intra-rater[tiab] OR intertester[tiab] OR inter-tester[tiab] OR intratester[tiab] OR intra-tester[tiab] OR interobserver[tiab] OR inter-observer[tiab] OR intraobserver[tiab] OR intra-observer[tiab] OR intertechnician[tiab] OR inter-technician[tiab] OR intratechnician[tiab] OR intra-technician[tiab] OR interexaminer[tiab] OR inter-examiner[tiab] OR intraexaminer[tiab] OR intra-examiner[tiab] OR interassay[tiab] OR inter-assay[tiab] OR intraassay[tiab] OR intra-assay[tiab] OR interindividual[tiab] OR inter-individual[tiab] OR intraindividual[tiab] OR intra-individual[tiab] OR interparticipant[tiab] OR inter-participant[tiab] OR intraparticipant[tiab] OR intra-participant[tiab] OR kappa[tiab] OR kappa’s[tiab] OR kappas[tiab] OR repeatab*[tw] OR ((replicab*[tw] OR repeated[tw]) AND (measure[tw] OR measures[tw] OR findings[tw] OR result[tw] OR results[tw] OR test[tw] OR tests[tw])) OR generaliza*[tiab] OR generalisa*[tiab] OR concordance[tiab] OR (intraclass[tiab] AND correlation*[tiab]) OR discriminative[tiab] OR "known group"[tiab] OR "factor analysis"[tiab] OR "factor analyses"[tiab] OR "factor structure"[tiab] OR "factor structures"[tiab] OR dimension*[tiab] OR subscale*[tiab] OR (multitrait[tiab] AND scaling[tiab] AND (analysis[tiab] OR analyses[tiab])) OR "item discriminant"[tiab] OR "interscale correlation*"[tiab] OR error[tiab] OR errors[tiab] OR "individual variability"[tiab] OR "interval variability"[tiab] OR "rate variability"[tiab] OR (variability[tiab] AND (analysis[tiab] OR values[tiab])) OR (uncertainty[tiab] AND (measurement[tiab] OR measuring[tiab])) OR "standard error of measurement"[tiab] OR sensitiv*[tiab] OR responsive*[tiab] OR (limit[tiab] AND detection[tiab]) OR "minimal detectable concentration"[tiab] OR interpretab*[tiab] OR ((minimal[tiab] OR minimally[tiab] OR clinical[tiab] OR clinically[tiab]) AND (important[tiab] OR significant[tiab] OR detectable[tiab]) AND (change[tiab] OR difference[tiab])) OR (small*[tiab] AND (real[tiab] OR detectable[tiab]) AND (change[tiab] OR difference[tiab])) OR "meaningful change"[tiab] OR "ceiling effect"[tiab] OR "floor effect"[tiab] OR "Item response model"[tiab] OR IRT[tiab] OR Rasch[tiab] OR "Differential item functioning"[tiab] OR DIF[tiab] OR "computer adaptive testing"[tiab] OR "item bank"[tiab] OR "cross-cultural equivalence"[tiab]))) NOT (("addresses"[Publication Type] OR "biography"[Publication Type] OR "case reports"[Publication Type] OR "comment"[Publication Type] OR "directory"[Publication Type] OR "editorial"[Publication Type] OR "festschrift"[Publication Type] OR "interview"[Publication Type] OR "lectures"[Publication Type] OR "legal cases"[Publication Type] OR "legislation"[Publication Type] OR "letter"[Publication Type] OR "news"[Publication Type] OR "newspaper article"[Publication Type] OR "patient education handout"[Publication Type] OR "popular works"[Publication Type] OR "congresses"[Publication Type] OR "consensus development conference"[Publication Type] OR "consensus development conference, nih"[Publication Type] OR "practice guideline"[Publication Type]) NOT ("animals"[MeSH Terms] NOT "humans"[MeSH Terms]))

Cleveland global quality of life
Hits: 6
Eligible: 0
(((Cleveland global quality of life) AND (anal fistula)) AND ((instrumentation[sh] OR methods[sh] OR "Validation Studies"[pt] OR "Comparative Study"[pt] OR "psychometrics"[MeSH] OR psychometr*[tiab] OR clinimetr*[tw] OR clinometr*[tw] OR "outcome assessment (health care)"[MeSH] OR "outcome assessment"[tiab] OR "outcome measure*"[tw] OR "observer variation"[MeSH] OR "observer variation"[tiab] OR "Health Status Indicators"[Mesh] OR "reproducibility of results"[MeSH] OR reproducib*[tiab] OR "discriminant analysis"[MeSH] OR reliab*[tiab] OR unreliab*[tiab] OR valid*[tiab] OR "coefficient of variation"[tiab] OR coefficient[tiab] OR homogeneity[tiab] OR homogeneous[tiab] OR "internal consistency"[tiab] OR (cronbach*[tiab] AND (alpha[tiab] OR alphas[tiab])) OR (item[tiab] AND (correlation*[tiab] OR selection*[tiab] OR reduction*[tiab])) OR agreement[tw] OR precision[tw] OR imprecision[tw] OR "precise values"[tw] OR test-retest[tiab] OR (test[tiab] AND retest[tiab]) OR (reliab*[tiab] AND (test[tiab] OR retest[tiab])) OR stability[tiab] OR interrater[tiab] OR inter-rater[tiab] OR intrarater[tiab] OR intra-rater[tiab] OR intertester[tiab] OR inter-tester[tiab] OR intratester[tiab] OR intra-tester[tiab] OR interobserver[tiab] OR inter-observer[tiab] OR intraobserver[tiab] OR intra-observer[tiab] OR intertechnician[tiab] OR inter-technician[tiab] OR intratechnician[tiab] OR intra-technician[tiab] OR interexaminer[tiab] OR inter-examiner[tiab] OR intraexaminer[tiab] OR intra-examiner[tiab] OR interassay[tiab] OR inter-assay[tiab] OR intraassay[tiab] OR intra-assay[tiab] OR interindividual[tiab] OR inter-individual[tiab] OR intraindividual[tiab] OR intra-individual[tiab] OR interparticipant[tiab] OR inter-participant[tiab] OR intraparticipant[tiab] OR intra-participant[tiab] OR kappa[tiab] OR kappa's[tiab] OR kappas[tiab] OR repeatab*[tw] OR ((replicab*[tw] OR repeated[tw]) AND (measure[tw] OR measures[tw] OR findings[tw] OR result[tw] OR results[tw] OR test[tw] OR tests[tw])) OR generaliza*[tiab] OR generalisa*[tiab] OR concordance[tiab] OR (intraclass[tiab] AND correlation*[tiab]) OR discriminative[tiab] OR "known group"[tiab] OR "factor analysis"[tiab] OR "factor analyses"[tiab] OR "factor structure"[tiab] OR "factor structures"[tiab] OR dimension*[tiab] OR subscale*[tiab] OR (multitrait[tiab] AND scaling[tiab] AND (analysis[tiab] OR analyses[tiab])) OR "item discriminant"[tiab] OR "interscale correlation*"[tiab] OR error[tiab] OR errors[tiab] OR "individual variability"[tiab] OR "interval variability"[tiab] OR "rate variability"[tiab] OR (variability[tiab] AND (analysis[tiab] OR values[tiab])) OR (uncertainty[tiab] AND (measurement[tiab] OR measuring[tiab])) OR "standard error of measurement"[tiab] OR sensitiv*[tiab] OR responsive*[tiab] OR (limit[tiab] AND detection[tiab]) OR "minimal detectable concentration"[tiab] OR interpretab*[tiab] OR ((minimal[tiab] OR minimally[tiab] OR clinical[tiab] OR clinically[tiab]) AND (important[tiab] OR significant[tiab] OR detectable[tiab]) AND (change[tiab] OR difference[tiab])) OR (small*[tiab] AND (real[tiab] OR detectable[tiab]) AND (change[tiab] OR difference[tiab])) OR "meaningful change"[tiab] OR "ceiling effect"[tiab] OR "floor effect"[tiab] OR "Item response model"[tiab] OR IRT[tiab] OR Rasch[tiab] OR "Differential item functioning"[tiab] OR DIF[tiab] OR "computer adaptive testing"[tiab] OR "item bank"[tiab] OR "cross-cultural equivalence"[tiab]))) NOT (("addresses"[Publication Type] OR "biography"[Publication Type] OR "case reports"[Publication Type] OR "comment"[Publication Type] OR "directory"[Publication Type] OR "editorial"[Publication Type] OR "festschrift"[Publication Type] OR "interview"[Publication Type] OR "lectures"[Publication Type] OR "legal cases"[Publication Type] OR "legislation"[Publication Type] OR "letter"[Publication Type] OR "news"[Publication Type] OR "newspaper article"[Publication Type] OR "patient education handout"[Publication Type] OR "popular works"[Publication Type] OR "congresses"[Publication Type] OR "consensus development conference"[Publication Type] OR "consensus development conference, nih"[Publication Type] OR "practice guideline"[Publication Type]) NOT ("animals"[MeSH Terms] NOT "humans"[MeSH Terms]))

Short Form-12 health survey (SF-12)
Hits: 9
Eligible: 0
((((Short Form-12 health survey) OR (SF-12)) AND (anal fistula)) AND ((instrumentation[sh] OR methods[sh] OR "Validation Studies"[pt] OR "Comparative Study"[pt] OR "psychometrics"[MeSH] OR psychometr*[tiab] OR clinimetr*[tw] OR clinometr*[tw] OR "outcome assessment (health care)"[MeSH] OR "outcome assessment"[tiab] OR "outcome measure*"[tw] OR "observer variation"[MeSH] OR "observer variation"[tiab] OR "Health Status Indicators"[Mesh] OR "reproducibility of results"[MeSH] OR reproducib*[tiab] OR "discriminant analysis"[MeSH] OR reliab*[tiab] OR unreliab*[tiab] OR valid*[tiab] OR "coefficient of variation"[tiab] OR coefficient[tiab] OR homogeneity[tiab] OR homogeneous[tiab] OR "internal consistency"[tiab] OR (cronbach*[tiab] AND (alpha[tiab] OR alphas[tiab])) OR (item[tiab] AND (correlation*[tiab] OR selection*[tiab] OR reduction*[tiab])) OR agreement[tw] OR precision[tw] OR imprecision[tw] OR "precise values"[tw] OR test-retest[tiab] OR (test[tiab] AND retest[tiab])

OR (reliab*[tiab] AND (test[tiab] OR retest[tiab])) OR stability[tiab] OR interrater[tiab] OR inter-rater[tiab] OR intrarater[tiab] OR intra-rater[tiab] OR intertester[tiab] OR inter-tester[tiab] OR intratester[tiab] OR intra-tester[tiab] OR interobserver[tiab] OR inter-observer[tiab] OR intraobserver[tiab] OR intra-observer[tiab] OR intertechnician[tiab] OR inter-technician[tiab] OR intratechnician[tiab] OR intra-technician[tiab] OR interexaminer[tiab] OR inter-examiner[tiab] OR intraexaminer[tiab] OR intra-examiner[tiab] OR interassay[tiab] OR inter-assay[tiab] OR intraassay[tiab] OR intra-assay[tiab] OR interindividual[tiab] OR inter-individual[tiab] OR intraindividual[tiab] OR intra-individual[tiab] OR interparticipant[tiab] OR inter-participant[tiab] OR intraparticipant[tiab] OR intra-participant[tiab] OR kappa[tiab] OR kappa’s[tiab] OR kappas[tiab] OR repeatab*[tw] OR ((replicab*[tw] OR repeated[tw]) AND (measure[tw] OR measures[tw] OR findings[tw] OR result[tw] OR results[tw] OR test[tw] OR tests[tw])) OR generaliza*[tiab] OR generalisa*[tiab] OR concordance[tiab] OR (intraclass[tiab] AND correlation*[tiab]) OR discriminative[tiab] OR "known group"[tiab] OR "factor analysis"[tiab] OR "factor analyses"[tiab] OR "factor structure"[tiab] OR "factor structures"[tiab] OR dimension*[tiab] OR subscale*[tiab] OR (multitrait[tiab] AND scaling[tiab] AND (analysis[tiab] OR analyses[tiab])) OR "item discriminant"[tiab] OR "interscale correlation*"[tiab] OR error[tiab] OR errors[tiab] OR "individual variability"[tiab] OR "interval variability"[tiab] OR "rate variability"[tiab] OR (variability[tiab] AND (analysis[tiab] OR values[tiab])) OR (uncertainty[tiab] AND (measurement[tiab] OR measuring[tiab])) OR "standard error of measurement"[tiab] OR sensitiv*[tiab] OR responsive*[tiab] OR (limit[tiab] AND detection[tiab]) OR "minimal detectable concentration"[tiab] OR interpretab*[tiab] OR ((minimal[tiab] OR minimally[tiab] OR clinical[tiab] OR clinically[tiab]) AND (important[tiab] OR significant[tiab] OR detectable[tiab]) AND (change[tiab] OR difference[tiab])) OR (small*[tiab] AND (real[tiab] OR detectable[tiab]) AND (change[tiab] OR difference[tiab])) OR "meaningful change"[tiab] OR "ceiling effect"[tiab] OR "floor effect"[tiab] OR "Item response model"[tiab] OR IRT[tiab] OR Rasch[tiab] OR "Differential item functioning"[tiab] OR DIF[tiab] OR "computer adaptive testing"[tiab] OR "item bank"[tiab] OR "cross-cultural equivalence"[tiab]))) NOT (("addresses"[Publication Type] OR "biography"[Publication Type] OR "case reports"[Publication Type] OR "comment"[Publication Type] OR "directory"[Publication Type] OR "editorial"[Publication Type] OR "festschrift"[Publication Type] OR "interview"[Publication Type] OR "lectures"[Publication Type] OR "legal cases"[Publication Type] OR "legislation"[Publication Type] OR "letter"[Publication Type] OR "news"[Publication Type] OR "newspaper article"[Publication Type] OR "patient education handout"[Publication Type] OR "popular works"[Publication Type] OR "congresses"[Publication Type] OR "consensus development conference"[Publication Type] OR "consensus development conference, nih"[Publication Type] OR "practice guideline"[Publication Type]) NOT ("animals"[MeSH Terms] NOT "humans"[MeSH Terms]))

Gastrointestinal Quality of Life Index (GIQLI)
Hits: 34
Eligible: 1 (10.1046/j.1365-2168.1998.00958.x)
(((Gastrointestinal Quality of Life Index) OR (GIQLI)) AND (anal fistula)) AND (((instrumentation[sh] OR methods[sh] OR "Validation Studies"[pt] OR "Comparative Study"[pt] OR "psychometrics"[MeSH] OR psychometr*[tiab] OR clinimetr*[tw] OR clinometr*[tw] OR "outcome assessment (health care)"[MeSH] OR "outcome assessment"[tiab] OR "outcome measure*"[tw] OR "observer variation"[MeSH] OR "observer variation"[tiab] OR "Health Status Indicators"[Mesh] OR "reproducibility of results"[MeSH] OR reproducib*[tiab] OR "discriminant analysis"[MeSH] OR reliab*[tiab] OR unreliab*[tiab] OR valid*[tiab] OR "coefficient of variation"[tiab] OR coefficient[tiab] OR homogeneity[tiab] OR homogeneous[tiab] OR "internal consistency"[tiab] OR (cronbach*[tiab] AND (alpha[tiab] OR alphas[tiab])) OR (item[tiab] AND (correlation*[tiab] OR selection*[tiab] OR reduction*[tiab])) OR agreement[tw] OR precision[tw] OR imprecision[tw] OR "precise values"[tw] OR test-retest[tiab] OR (test[tiab] AND retest[tiab]) OR (reliab*[tiab] AND (test[tiab] OR retest[tiab])) OR stability[tiab] OR interrater[tiab] OR inter-rater[tiab] OR intrarater[tiab] OR intra-rater[tiab] OR intertester[tiab] OR inter-tester[tiab] OR intratester[tiab] OR intra-tester[tiab] OR interobserver[tiab] OR inter-observer[tiab] OR intraobserver[tiab] OR intra-observer[tiab] OR intertechnician[tiab] OR inter-technician[tiab] OR intratechnician[tiab] OR intra-technician[tiab] OR interexaminer[tiab] OR inter-examiner[tiab] OR intraexaminer[tiab] OR intra-examiner[tiab] OR interassay[tiab] OR inter-assay[tiab] OR intraassay[tiab] OR intra-assay[tiab] OR interindividual[tiab] OR inter-individual[tiab] OR intraindividual[tiab] OR intra-individual[tiab] OR interparticipant[tiab] OR inter-participant[tiab] OR intraparticipant[tiab] OR intra-participant[tiab] OR kappa[tiab] OR kappa’s[tiab] OR kappas[tiab] OR repeatab*[tw] OR ((replicab*[tw] OR repeated[tw]) AND (measure[tw] OR measures[tw] OR findings[tw] OR result[tw] OR results[tw] OR test[tw] OR tests[tw])) OR generaliza*[tiab] OR generalisa*[tiab] OR concordance[tiab] OR (intraclass[tiab] AND correlation*[tiab]) OR discriminative[tiab] OR "known group"[tiab] OR "factor analysis"[tiab] OR "factor analyses"[tiab] OR "factor structure"[tiab] OR "factor structures"[tiab] OR

dimension*[tiab] OR subscale*[tiab] OR (multitrait[tiab] AND scaling[tiab] AND (analysis[tiab] OR analyses[tiab])) OR "item discriminant"[tiab] OR "interscale correlation*"[tiab] OR error[tiab] OR errors[tiab] OR "individual variability"[tiab] OR "interval variability"[tiab] OR "rate variability"[tiab] OR (variability[tiab] AND (analysis[tiab] OR values[tiab])) OR (uncertainty[tiab] AND (measurement[tiab] OR measuring[tiab])) OR "standard error of measurement"[tiab] OR sensitiv*[tiab] OR responsive*[tiab] OR (limit[tiab] AND detection[tiab]) OR "minimal detectable concentration"[tiab] OR interpretab*[tiab] OR ((minimal[tiab] OR minimally[tiab] OR clinical[tiab] OR clinically[tiab]) AND (important[tiab] OR significant[tiab] OR detectable[tiab]) AND (change[tiab] OR difference[tiab])) OR (small*[tiab] AND (real[tiab] OR detectable[tiab]) AND (change[tiab] OR difference[tiab])) OR "meaningful change"[tiab] OR "ceiling effect"[tiab] OR "floor effect"[tiab] OR "Item response model"[tiab] OR IRT[tiab] OR Rasch[tiab] OR "Differential item functioning"[tiab] OR DIF[tiab] OR "computer adaptive testing"[tiab] OR "item bank"[tiab] OR "cross-cultural equivalence"[tiab]))) NOT (("addresses"[Publication Type] OR "biography"[Publication Type] OR "case reports"[Publication Type] OR "comment"[Publication Type] OR "directory"[Publication Type] OR "editorial"[Publication Type] OR "festschrift"[Publication Type] OR "interview"[Publication Type] OR "lectures"[Publication Type] OR "legal cases"[Publication Type] OR "legislation"[Publication Type] OR "letter"[Publication Type] OR "news"[Publication Type] OR "newspaper article"[Publication Type] OR "patient education handout"[Publication Type] OR "popular works"[Publication Type] OR "congresses"[Publication Type] OR "consensus development conference"[Publication Type] OR "consensus development conference, nih"[Publication Type] OR "practice guideline"[Publication Type]) NOT ("animals"[MeSH Terms] NOT "humans"[MeSH Terms])))

Visual Analogue Scale (VAS)
Hits: 60
Eligible: 0
((((Visual Analogue Scale) OR (VAS)) AND (anal fistula)) AND ((instrumentation[sh] OR methods[sh] OR "Validation Studies"[pt] OR "Comparative Study"[pt] OR "psychometrics"[MeSH] OR psychometr*[tiab] OR clinimetr*[tw] OR clinometr*[tw] OR "outcome assessment (health care)"[MeSH] OR "outcome assessment"[tiab] OR "outcome measure*"[tw] OR "observer variation"[MeSH] OR "observer variation"[tiab] OR "Health Status Indicators"[Mesh] OR "reproducibility of results"[MeSH] OR reproducib*[tiab] OR "discriminant analysis"[MeSH] OR reliab*[tiab] OR unreliab*[tiab] OR valid*[tiab] OR "coefficient of variation"[tiab] OR coefficient[tiab] OR homogeneity[tiab] OR homogeneous[tiab] OR "internal consistency"[tiab] OR (cronbach*[tiab] AND (alpha[tiab] OR alphas[tiab])) OR (item[tiab] AND (correlation*[tiab] OR selection*[tiab] OR reduction*[tiab])) OR agreement[tw] OR precision[tw] OR imprecision[tw] OR "precise values"[tw] OR test-retest[tiab] OR (test[tiab] AND retest[tiab]) OR (reliab*[tiab] AND (test[tiab] OR retest[tiab])) OR stability[tiab] OR interrater[tiab] OR inter-rater[tiab] OR intrarater[tiab] OR intra-rater[tiab] OR intertester[tiab] OR inter-tester[tiab] OR intratester[tiab] OR intra-tester[tiab] OR interobserver[tiab] OR inter-observer[tiab] OR intraobserver[tiab] OR intra-observer[tiab] OR intertechnician[tiab] OR inter-technician[tiab] OR intratechnician[tiab] OR intra-technician[tiab] OR interexaminer[tiab] OR inter-examiner[tiab] OR intraexaminer[tiab] OR intra-examiner[tiab] OR interassay[tiab] OR inter-assay[tiab] OR intraassay[tiab] OR intra-assay[tiab] OR interindividual[tiab] OR inter-individual[tiab] OR intraindividual[tiab] OR intra-individual[tiab] OR interparticipant[tiab] OR inter-participant[tiab] OR intraparticipant[tiab] OR intra-participant[tiab] OR kappa[tiab] OR kappa's[tiab] OR kappas[tiab] OR repeatab*[tw] OR ((replicab*[tw] OR repeated[tw]) AND (measure[tw] OR measures[tw] OR findings[tw] OR result[tw] OR results[tw] OR test[tw] OR tests[tw])) OR generaliza*[tiab] OR generalisa*[tiab] OR concordance[tiab] OR (intraclass[tiab] AND correlation*[tiab]) OR discriminative[tiab] OR "known group"[tiab] OR "factor analysis"[tiab] OR "factor analyses"[tiab] OR "factor structure"[tiab] OR "factor structures"[tiab] OR dimension*[tiab] OR subscale*[tiab] OR (multitrait[tiab] AND scaling[tiab] AND (analysis[tiab] OR analyses[tiab])) OR "item discriminant"[tiab] OR "interscale correlation*"[tiab] OR error[tiab] OR errors[tiab] OR "individual variability"[tiab] OR "interval variability"[tiab] OR "rate variability"[tiab] OR (variability[tiab] AND (analysis[tiab] OR values[tiab])) OR (uncertainty[tiab] AND (measurement[tiab] OR measuring[tiab])) OR "standard error of measurement"[tiab] OR sensitiv*[tiab] OR responsive*[tiab] OR (limit[tiab] AND detection[tiab]) OR "minimal detectable concentration"[tiab] OR interpretab*[tiab] OR ((minimal[tiab] OR minimally[tiab] OR clinical[tiab] OR clinically[tiab]) AND (important[tiab] OR significant[tiab] OR detectable[tiab]) AND (change[tiab] OR difference[tiab])) OR (small*[tiab] AND (real[tiab] OR detectable[tiab]) AND (change[tiab] OR difference[tiab])) OR "meaningful change"[tiab] OR "ceiling effect"[tiab] OR "floor effect"[tiab] OR "Item response model"[tiab] OR IRT[tiab] OR Rasch[tiab] OR "Differential item functioning"[tiab] OR DIF[tiab] OR "computer adaptive testing"[tiab] OR "item bank"[tiab] OR "cross-cultural equivalence"[tiab]))) NOT (("addresses"[Publication Type] OR "biography"[Publication Type] OR "case reports"[Publication Type] OR "comment"[Publication Type] OR

"directory"[Publication Type] OR "editorial"[Publication Type] OR "festschrift"[Publication Type] OR "interview"[Publication Type] OR "lectures"[Publication Type] OR "legal cases"[Publication Type] OR "legislation"[Publication Type] OR "letter"[Publication Type] OR "news"[Publication Type] OR "newspaper article"[Publication Type] OR "patient education handout"[Publication Type] OR "popular works"[Publication Type] OR "congresses"[Publication Type] OR "consensus development conference"[Publication Type] OR "consensus development conference, nih"[Publication Type] OR "practice guideline"[Publication Type]) NOT ("animals"[MeSH Terms] NOT "humans"[MeSH Terms]))

Fecal Incontinence Severity Index (FISI)
Hits: 52
Eligible: 0
((((Fecal Incontinence Severity Index) OR (FISI)) AND (anal fistula)) AND ((instrumentation[sh] OR methods[sh] OR "Validation Studies"[pt] OR "Comparative Study"[pt] OR "psychometrics"[MeSH] OR psychometr*[tiab] OR clinimetr*[tw] OR clinometr*[tw] OR "outcome assessment (health care)"[MeSH] OR "outcome assessment"[tiab] OR "outcome measure*"[tw] OR "observer variation"[MeSH] OR "observer variation"[tiab] OR "Health Status Indicators"[Mesh] OR "reproducibility of results"[MeSH] OR reproducib*[tiab] OR "discriminant analysis"[MeSH] OR reliab*[tiab] OR unreliab*[tiab] OR valid*[tiab] OR "coefficient of variation"[tiab] OR coefficient[tiab] OR homogeneity[tiab] OR homogeneous[tiab] OR "internal consistency"[tiab] OR (cronbach*[tiab] AND (alpha[tiab] OR alphas[tiab])) OR (item[tiab] AND (correlation*[tiab] OR selection*[tiab] OR reduction*[tiab])) OR agreement[tw] OR precision[tw] OR imprecision[tw] OR "precise values"[tw] OR test-retest[tiab] OR (test[tiab] AND retest[tiab]) OR (reliab*[tiab] AND (test[tiab] OR retest[tiab])) OR stability[tiab] OR interrater[tiab] OR inter-rater[tiab] OR intrarater[tiab] OR intra-rater[tiab] OR intertester[tiab] OR inter-tester[tiab] OR intratester[tiab] OR intra-tester[tiab] OR interobserver[tiab] OR inter-observer[tiab] OR intraobserver[tiab] OR intra-observer[tiab] OR intertechnician[tiab] OR inter-technician[tiab] OR intratechnician[tiab] OR intra-technician[tiab] OR interexaminer[tiab] OR inter-examiner[tiab] OR intraexaminer[tiab] OR intra-examiner[tiab] OR interassay[tiab] OR inter-assay[tiab] OR intraassay[tiab] OR intra-assay[tiab] OR interindividual[tiab] OR inter-individual[tiab] OR intraindividual[tiab] OR intra-individual[tiab] OR interparticipant[tiab] OR inter-participant[tiab] OR intraparticipant[tiab] OR intra-participant[tiab] OR kappa[tiab] OR kappa’s[tiab] OR kappas[tiab] OR repeatab*[tw] OR ((replicab*[tw] OR repeated[tw]) AND (measure[tw] OR measures[tw] OR findings[tw] OR result[tw] OR results[tw] OR test[tw] OR tests[tw])) OR generaliza*[tiab] OR generalisa*[tiab] OR concordance[tiab] OR (intraclass[tiab] AND correlation*[tiab]) OR discriminative[tiab] OR "known group"[tiab] OR "factor analysis"[tiab] OR "factor analyses"[tiab] OR "factor structure"[tiab] OR "factor structures"[tiab] OR dimension*[tiab] OR subscale*[tiab] OR (multitrait[tiab] AND scaling[tiab] AND (analysis[tiab] OR analyses[tiab])) OR "item discriminant"[tiab] OR "interscale correlation*"[tiab] OR error[tiab] OR errors[tiab] OR "individual variability"[tiab] OR "interval variability"[tiab] OR "rate variability"[tiab] OR (variability[tiab] AND (analysis[tiab] OR values[tiab])) OR (uncertainty[tiab] AND (measurement[tiab] OR measuring[tiab])) OR "standard error of measurement"[tiab] OR sensitiv*[tiab] OR responsive*[tiab] OR (limit[tiab] AND detection[tiab]) OR "minimal detectable concentration"[tiab] OR interpretab*[tiab] OR ((minimal[tiab] OR minimally[tiab] OR clinical[tiab] OR clinically[tiab]) AND (important[tiab] OR significant[tiab] OR detectable[tiab]) AND (change[tiab] OR difference[tiab])) OR (small*[tiab] AND (real[tiab] OR detectable[tiab]) AND (change[tiab] OR difference[tiab])) OR "meaningful change"[tiab] OR "ceiling effect"[tiab] OR "floor effect"[tiab] OR "Item response model"[tiab] OR IRT[tiab] OR Rasch[tiab] OR "Differential item functioning"[tiab] OR DIF[tiab] OR "computer adaptive testing"[tiab] OR "item bank"[tiab] OR "cross-cultural equivalence"[tiab]))) NOT (("addresses"[Publication Type] OR "biography"[Publication Type] OR "case reports"[Publication Type] OR "comment"[Publication Type] OR "directory"[Publication Type] OR "editorial"[Publication Type] OR "festschrift"[Publication Type] OR "interview"[Publication Type] OR "lectures"[Publication Type] OR "legal cases"[Publication Type] OR "legislation"[Publication Type] OR "letter"[Publication Type] OR "news"[Publication Type] OR "newspaper article"[Publication Type] OR "patient education handout"[Publication Type] OR "popular works"[Publication Type] OR "congresses"[Publication Type] OR "consensus development conference"[Publication Type] OR "consensus development conference, nih"[Publication Type] OR "practice guideline"[Publication Type]) NOT ("animals"[MeSH Terms] NOT "humans"[MeSH Terms]))

Quality of Life in patients with Anal Fistula Questionnaire (QoLAF-Q)
Hits: 85
Eligible: 2 (10.1097/DCR.0000000000000877 & 10.3390/clinpract12040066.)

((((Quality of Life in patients with Anal Fistula Questionnaire) OR (QoLAF-Q)) AND (anal fistula)) AND ((instrumentation[sh] OR methods[sh] OR "Validation Studies"[pt] OR "Comparative Study"[pt] OR "psychometrics"[MeSH] OR psychometr*[tiab] OR clinimetr*[tw] OR clinometr*[tw] OR "outcome assessment (health care)"[MeSH] OR "outcome assessment"[tiab] OR "outcome measure*"[tw] OR "observer variation"[MeSH] OR "observer variation"[tiab] OR "Health Status Indicators"[Mesh] OR "reproducibility of results"[MeSH] OR reproducib*[tiab] OR "discriminant analysis"[MeSH] OR reliab*[tiab] OR unreliab*[tiab] OR valid*[tiab] OR "coefficient of variation"[tiab] OR coefficient[tiab] OR homogeneity[tiab] OR homogeneous[tiab] OR "internal consistency"[tiab] OR (cronbach*[tiab] AND (alpha[tiab] OR alphas[tiab])) OR (item[tiab] AND (correlation*[tiab] OR selection*[tiab] OR reduction*[tiab])) OR agreement[tw] OR precision[tw] OR imprecision[tw] OR "precise values"[tw] OR test-retest[tiab] OR (test[tiab] AND retest[tiab]) OR (reliab*[tiab] AND (test[tiab] OR retest[tiab])) OR stability[tiab] OR interrater[tiab] OR inter-rater[tiab] OR intrarater[tiab] OR intra-rater[tiab] OR intertester[tiab] OR inter-tester[tiab] OR intratester[tiab] OR intra-tester[tiab] OR interobserver[tiab] OR inter-observer[tiab] OR intraobserver[tiab] OR intra-observer[tiab] OR intertechnician[tiab] OR inter-technician[tiab] OR intratechnician[tiab] OR intra-technician[tiab] OR interexaminer[tiab] OR inter-examiner[tiab] OR intraexaminer[tiab] OR intra-examiner[tiab] OR interassay[tiab] OR inter-assay[tiab] OR intraassay[tiab] OR intra-assay[tiab] OR interindividual[tiab] OR inter-individual[tiab] OR intraindividual[tiab] OR intra-individual[tiab] OR interparticipant[tiab] OR inter-participant[tiab] OR intraparticipant[tiab] OR intra-participant[tiab] OR kappa[tiab] OR kappa's[tiab] OR kappas[tiab] OR repeatab*[tw] OR ((replicab*[tw] OR repeated[tw]) AND (measure[tw] OR measures[tw] OR findings[tw] OR result[tw] OR results[tw] OR test[tw] OR tests[tw])) OR generaliza*[tiab] OR generalisa*[tiab] OR concordance[tiab] OR (intraclass[tiab] AND correlation*[tiab]) OR discriminative[tiab] OR "known group"[tiab] OR "factor analysis"[tiab] OR "factor analyses"[tiab] OR "factor structure"[tiab] OR "factor structures"[tiab] OR dimension*[tiab] OR subscale*[tiab] OR (multitrait[tiab] AND scaling[tiab] AND (analysis[tiab] OR analyses[tiab])) OR "item discriminant"[tiab] OR "interscale correlation*"[tiab] OR error[tiab] OR errors[tiab] OR "individual variability"[tiab] OR "interval variability"[tiab] OR "rate variability"[tiab] OR (variability[tiab] AND (analysis[tiab] OR values[tiab])) OR (uncertainty[tiab] AND (measurement[tiab] OR measuring[tiab])) OR "standard error of measurement"[tiab] OR sensitiv*[tiab] OR responsive*[tiab] OR (limit[tiab] AND detection[tiab]) OR "minimal detectable concentration"[tiab] OR interpretab*[tiab] OR ((minimal[tiab] OR minimally[tiab] OR clinical[tiab] OR clinically[tiab]) AND (important[tiab] OR significant[tiab] OR detectable[tiab]) AND (change[tiab] OR difference[tiab])) OR (small*[tiab] AND (real[tiab] OR detectable[tiab]) AND (change[tiab] OR difference[tiab])) OR "meaningful change"[tiab] OR "ceiling effect"[tiab] OR "floor effect"[tiab] OR "Item response model"[tiab] OR IRT[tiab] OR Rasch[tiab] OR "Differential item functioning"[tiab] OR DIF[tiab] OR "computer adaptive testing"[tiab] OR "item bank"[tiab] OR "cross-cultural equivalence"[tiab]))) NOT (("addresses"[Publication Type] OR "biography"[Publication Type] OR "case reports"[Publication Type] OR "comment"[Publication Type] OR "directory"[Publication Type] OR "editorial"[Publication Type] OR "festschrift"[Publication Type] OR "interview"[Publication Type] OR "lectures"[Publication Type] OR "legal cases"[Publication Type] OR "legislation"[Publication Type] OR "letter"[Publication Type] OR "news"[Publication Type] OR "newspaper article"[Publication Type] OR "patient education handout"[Publication Type] OR "popular works"[Publication Type] OR "congresses"[Publication Type] OR "consensus development conference"[Publication Type] OR "consensus development conference, nih"[Publication Type] OR "practice guideline"[Publication Type]) NOT ("animals"[MeSH Terms] NOT "humans"[MeSH Terms]))

2.: *All instrument cards created in Phase 2.*

**Instrument name**	**Perianal Fıstula Disease Severity Scores**
**Acronym:**	PAF/PAD severity scores
**Domains assessed:**	(1) Clinical fistula healing
**Description:**	PAD severity scores based on a 5 category scoring system. Used for Crohn's fistula. Scores were determined by the surgeon and recorded at the time of surgery and at each subsequent postoperative visit.
**Number of items:**	Total questions: 1
**Estimated time to complete:**	1-2 minute(s)
**Administer to:**	Patients
**Require specially trained tester:**	No
**Mode of administration:**	In-person, Phone, Mail
**License/Purchasing tests/Contact and copyright information/Website:**	This measurement scale has not been licensed.
**Current licensing/Equipment costs:**	This measurement scale has no licensing fees.
**Equipment required/useful:**	Survey form and pen
**Number of published cryptoglandular anal fistula publications using instrument (2008 – 2022):**	1 publication used this instrument as a specific measure of healing
**Highest COSMIN rating:**	No evaluation was completed in validating this measurement scale in a population of cryptoglandular perianal fistula patients. Therefore no comment can be made in this regard.
**Additional comments:**	This measurement scale was created by the authors of the original study regarding Crohn's fistula. It has been used in one published study regarding cryptoglandular fistula.
**Online example:**	Moy J, Bodzin J. Carbon dioxide laser ablation of perianal fistulas in patients with Crohn's disease: experience with 27 patients. Am J Surg. 2006 Mar;191(3):424-7. doi: 10.1016/j.amjsurg.2005.10.050. PMID: 16490560.

**Instrument name**	**Perianal Disease Activity Index**
**Acronym:**	PDAI
**Domains assessed:**	(1) Healing
**Description:**	This index was created to independently and objectively evaluate anal lesions on CD. The evaluation items consisted of five items, and in addition to the clinical evaluation items, HR-QoL was evaluated by the patient regarding pain and restriction of sexual activity.
**Number of items:**	Total questions: 5
**Estimated time to complete:**	3-5 minute(s)
**Administer to:**	Patients
**Require specially trained tester:**	No
**Mode of administration:**	In-person
**License/Purchasing tests/Contact and copyright information/Website:**	This measurement scale has not been licensed.
**Current licensing/Equipment costs:**	This measurement scale has no licensing fees.
**Equipment required/useful:**	Survey form and pen
**Number of published cryptoglandular anal fistula publications using instrument (2008 – 2022):**	1 publication used this instrument as a specific measure of healing
**Highest COSMIN rating:**	No evaluation was completed in validating this measurement scale in a population of cryptoglandular perianal fistula patients. Therefore no comment can be made in this regard.

**Additional comments:**	This measurement scale was created by the authors of the original study regarding Crohn's fistula. It has been used in one published study regarding cryptoglandular fistula.
**Online example:**	Irvine EJ. Usual therapy improves perianal Crohn's disease as measured by a new disease activity index. McMaster IBD Study Group. J Clin Gastroenterol 1995;20:27–32<br>Sandborn WJ et al. (2002) A review of activity indices and efficacy endpoints for clinical trials of medical therapy in adults with Crohn's disease. Gastroenterology 122: 512–530

**Instrument name**	**Fecal Incontinence Quality of Life Scale**
**Acronym:**	FIQL or Rockwood scale
**Domains assessed:**	Quality of Life
**Description:**	The Fecal Incontinence Quality of Life Scale is composed of a total of 29 items; these items form four scales: Lifestyle (10 items), Coping/Behavior (9 items), Depression/Self-Perception (7 items), and Embarrassment (3 items).
**Number of items:**	29 items
**Estimated time to complete:**	N/A
**Administer to:**	Patients
**Require specially trained tester:**	No
**Mode of administration:**	In-person, Online
**License/Purchasing tests/Contact and copyright information/Website:**	This measurement scale has not been licensed.
**Current licensing/Equipment costs:**	This measurement scale has no licensing fees.
**Equipment required/useful:**	In person: Survey form and pen<br>Online: Access to a computer or phone with internet access
**Number of published cryptoglandular anal fistula publications using instrument (2008 – 2022):**	Used 9 times in literature
**Highest COSMIN rating:**	No evaluation completed in validating this measurement scale in a population of cryptoglandular perianal fistula patients. Therefore no comment can be made in this regard.
**Additional comments:**	This measurement scale was created to evaluate quality of life in patients suffering with fecal incontinence
**Online example:**	Rockwood TH, Church JM, Fleshman JW, Kane RL, Mavrantonis C, Thorson AG, Wexner SD, Bliss D, Lowry AC. Fecal Incontinence Quality of Life Scale: quality of life instrument for patients with fecal incontinence. Dis Colon Rectum. 2000 Jan;43(1):9-16; discussion 16-7. doi: 10.1007/BF02237236. PMID: 10813117.

**Instrument name**	**Fecal Incontinence Severity Index**
**Acronym:**	FISI
**Domains assessed:**	Quality of Life, Incontinence
**Description:**	The fecal incontinence severity index helps quantify the impact of adult incontinence leakage on quality of life. It describes the severity of different types of incontinence for bowel contents.
**Number of items:**	4
**Estimated time to complete:**	< 1 minute
**Administer to:**	Patients
**Require specially trained tester:**	No
**Mode of administration:**	In-person, Online
**License/Purchasing tests/Contact and copyright information/Website:**	This measurement scale has not been licensed.
**Current licensing/Equipment costs:**	This measurement scale has no licensing fees.
**Equipment required/useful:**	In person: Survey form and pen

	Online: Access to a computer or phone with internet access
**Number of published cryptoglandular anal fistula publications using instrument (2008 – 2022):**	Used 1 times in literature for the purpose of scoring Quality of Life
**Highest COSMIN rating:**	No evaluation was completed in validating this measurement scale in a population of cryptoglandular perianal fistula patients. Therefore no comment can be made in this regard.
**Additional comments:**	The final result is the sum of all points and varies from 0 to 61, where the higher the score, the higher the perceived severity of the fecal incontinence.<br>The original study by Rockwood et al. does not provide a specific cut off and the rule of thumb is the one introduced above.<br>Patients who score closer to 61 are likely to have their quality of life severely impacted by the fecal incontinence they suffer from.<br>A subsequent study by Cavanaugh et al. found that FISI scores above 30 are more likely to be associated with an impaired quality of life than scores of 30 and below.
**Online example:**	Rockwood TH, Church JM, Fleshman JW, Kane RL, Mavrantonis C, Thorson AG, Wexner SD, Bliss D, Lowry AC. Patient and surgeon ranking of the severity of symptoms associated with fecal incontinence: the fecal incontinence severity index. Dis Colon Rectum. 1999; 42(12):1525-32.

**Instrument name**	**Quality of Life Anal Fistula Questionnaire**
**Acronym:**	QoLAF-Q
**Domains assessed:**	(2) Fistula symptoms & Quality of Life
**Description:**	The Quality of Life Anal Fistula Questionnaire (QoLAF-Q) was developed to assess QoL in patients with an anal fistula, in order to aid decision making in treatment strategies.
**Number of items:**	Total questions: 14
**Estimated time to complete:**	N/A
**Administer to:**	Patients
**Require specially trained tester:**	No
**Mode of administration:**	In-person, Online
**License/Purchasing tests/Contact and copyright information/Website:**	This measurement scale has not been licensed.
**Current licensing/Equipment costs:**	This measurement scale has no licensing fees.
**Equipment required/useful:**	Survey form and pen, Computer
**Number of published cryptoglandular anal fistula publications using instrument (2008 – 2022):**	1 publication used this instrument as a specific measure of Quality of Life
**Highest COSMIN rating:**	Evaluation was completed in validating this measurement scale in a population of cryptoglandular perianal fistula patients.<br><br>Relevance rating: *very low*<br>Comprehensiveness rating: *very low*<br>Comprehensibility rating: *very low*<br>Content validity rating: *very low*<br><br>Iqbal N, Shah R, Alrubaiy L, Tozer P. Do Patient-Reported Quality-of-Life (QoL) Scales Provide an Adequate Assessment of Patients with Cryptoglandular Anal Fistulae? A Systematic Review of Measurement Instruments and Their Content Validity. Clin Pract. 2022 Aug 15;12(4):628-639. doi: 10.3390/clinpract12040066. PMID: 36005069; PMCID: PMC9406553.
**Additional comments:**	This questionnaire specifically measures quality of life in people with anal fistula and its score range is the following: zero impact=14 points, limited impact=15 to 28 points, moderate impact=29 to 42 points, high impact=43 to 56 points, and very high impact=57 to 70 points.

**Online example:**	Ferrer-Márquez M, Espínola-Cortés N, Reina-Duarte Á, Granero-Molina J, Fernández-Sola C, Hernández-Padilla JM. Análisis y descripción de la calidad de vida específica en pacientes con fístula anal. Cir Esp. 2018;96:213–220. Ferrer-Márquez, M, Espínola-Cortés, N; Reina-Duarte, A; Granero-Molina, J; Fernández-Sola, C; Hernández-Padilla, J. Design and Psychometric Evaluation of the Quality of Life in Patients With Anal Fistula Questionnaire. Diseases of the Colon & Rectum: October 2017 - Volume 60 - Issue 10 - p 1083-1091 doi: 10.1097/DCR.0000000000000877

**Instrument name**	**Gastrointestinal Quality of Life Index**
**Acronym:**	GIQLI
**Domains assessed:**	(1) Quality of Life
**Description:**	It includes 36 items divided between five domains: symptoms, physical dysfunction, emotional dysfunctions, social dysfunction, and a single-item question on the effect of medical treatment. Each domain includes 4 to 19 items. It has been applied in patients with various gastrointestinal disorders, including gastroesophageal reflux disease, gallstone related symptoms, and irritable bowel syndrome.
**Number of items:**	Total questions: 36
**Estimated time to complete:**	5 minutes
**Administer to:**	Patients
**Require specially trained tester:**	No
**Mode of administration:**	In-person, Online
**License/Purchasing tests/Contact and copyright information/Website:**	This measurement scale has not been licensed.
**Current licensing/Equipment costs:**	This measurement scale has no licensing fees.
**Equipment required/useful:**	In person: Survey form and pen<br>Online: Access to a computer or phone with internet access
**Number of published cryptoglandular anal fistula publications using instrument (2008 – 2022):**	Used 1 times in literature for the purpose of scoring Quality of Life
**Highest COSMIN rating:**	No evaluation was completed in validating this measurement scale in a population of cryptoglandular perianal fistula patients. Therefore no comment can be made in this regard.
**Additional comments:**	The GIQLI was developed on patients with benign and malignant GI disorders including anorectal disease, but there are no specific details regarding their diagnoses. Items in the symptom domain focus on abdominal pain, bloating, and bowel movements, all of which are unrelated to cryptoglandular anal fistulae. Its use in patients with benign anorectal disorders has been studied, including a cohort of 22 fistula patients, finding that QoL as measured by the instrument did not differ significantly in fistula patients, those with haemorrhoids, or a symptomatic rectocele when compared to age-matched controls. The study suggested that this could be explained by patient adaptation to symptoms. However, it could be argued that this finding is largely artefactual, since these patients would be unlikely to demonstrate poor QoL if it was measured using questions about symptoms that are largely irrelevant to their disease process.
**Online example:**	M Sailer and others, Quality of life in patients with benign anorectal disorders, British Journal of Surgery, Volume 85, Issue 12, December 1998, Pages 1716–1719, https://doi.org/10.1046/j.1365-2168.1998.00958.x

**Instrument name**	**36-Item Short Form Health Survey**
**Acronym:**	SF-36 / MOS SF-36 / RAND-36
**Domains assessed:**	Quality of life
**Description:**	The SF-36 is a measure of health status and is commonly used in health economics as a variable in the quality-adjusted life year calculation to determine the cost-effectiveness of a health treatment.

**Number of items:**	36
**Estimated time to complete:**	7-8 minutes
**Administer to:**	Patients
**Require specially trained tester:**	No
**Mode of administration:**	In person, Online
**License/Purchasing tests/Contact and copyright information/Website:**	This measurement scale has not been licensed.
**Current licensing/Equipment costs:**	This measurement scale has no licensing fees.
**Equipment required/useful:**	In person: Survey form and pen<br>Online: Access to a computer or phone with internet access
**Number of published cryptoglandular anal fistula publications using instrument (2008 – 2022):**	Used 8 times in literature for the purpose of scoring Quality of Life
**Highest COSMIN rating:**	No evaluation was completed in validating this measurement scale in a population of cryptoglandular perianal fistula patients. Therefore no comment can be made in this regard.
**Additional comments:**	The SF-36 is a validated HRQOL tool that can be used in a wide spectrum of medical conditions. It has been translated and validated in many languages and cultures. The SF-36 covers eight different dimensions of HRQOL: physical functioning, social functioning, role limitations due to physical functioning (role functioning—physical), bodily gain, general mental health, role limitations due to emotional functioning (role functioning—emotional), vitality (energy and fatigue), and general health perception. Scores can be summed together from all domains with differing weightings to contribute to two summary scores, a physical component score and a mental component score.
**Online example:**	Owen HA, Buchanan GN, Schizas A, Cohen R, Williams AB. Quality of life with anal fistula. Ann R Coll Surg Engl. 2016 May;98(5):334-8. doi: 10.1308/rcsann.2016.0136. PMID: 27087327; PMCID: PMC5227050.

**Instrument name**	**12-item Short Form Health Survey**
**Acronym:**	SF-12 / MOS-12/ RAND-12
**Domains assessed:**	Quality of Life
**Description:**	The Short Form (12) Health Survey is a 12-item, patient-reported survey of patient health.
**Number of items:**	12
**Estimated time to complete:**	4-5 minutes
**Administer to:**	Patients
**Require specially trained tester:**	No
**Mode of administration:**	In person, Online
**License/Purchasing tests/Contact and copyright information/Website:**	QualityMetric holds the licensing rights for this measurement scale.
**Current licensing/Equipment costs:**	The utilization of this scale is accompanied by applicable licensing fees.
**Equipment required/useful:**	In person: Survey form and pen<br>Online: Access to a computer or phone with internet access
**Number of published cryptoglandular anal fistula publications using instrument (2008 – 2022):**	Used 2 times in literature for the purpose of scoring Quality of Life
**Highest COSMIN rating:**	No evaluation was completed in validating this measurement scale in a population of cryptoglandular perianal fistula patients. Therefore no comment can be made in this regard.
**Additional comments:**	It is a reduced size version of the SF-36, and is widely used since it produces similar results for physical and mental health scores with far less respondent burden for producing scores of overall mental and physical well-being.
**Online example:**	Ferrer-Márquez M, Espínola-Cortés N, Reina-Duarte Á, Granero-Molina J, Fernández-Sola C, Hernández-Padilla JM. Análisis y descripción de la calidad de vida específica

	en pacientes con fístula anal. Cir Esp. 2018;96:213–220. Ferrer-Márquez, M, Espínola-Cortés, N; Reina-Duarte, A; Granero-Molina, J; Fernández-Sola, C; Hernández-Padilla, J. Design and Psychometric Evaluation of the Quality of Life in Patients With Anal Fistula Questionnaire. Diseases of the Colon & Rectum: October 2017 - Volume 60 - Issue 10 - p 1083-1091 doi: 10.1097/DCR.0000000000000877

**Instrument name**	**EuroQol 5D**
**Acronym:**	EQ-5D
**Domains assessed:**	Quality of Life
**Description:**	The EQ-5D is a widely used health-related quality of life questionnaire that assesses an individual's overall well-being across multiple dimensions including mobility, self-care, usual activities, pain/discomfort, and anxiety/depression.
**Number of items:**	6 items
**Estimated time to complete:**	5-10 minutes
**Administer to:**	Patients
**Require specially trained tester:**	No
**Mode of administration:**	In-person, Online
**License/Purchasing tests/Contact and copyright information/Website:**	EuroQol holds the licensing rights for this measurement scale.
**Current licensing/Equipment costs:**	This measurement scale has no licensing fees.
**Equipment required/useful:**	In person: Survey form and pen<br>Online: Access to a computer or phone with internet access
**Number of published cryptoglandular anal fistula publications using instrument (2008 – 2022):**	Used 5 times in literature for the purpose of scoring Quality of Life
**Highest COSMIN rating:**	No evaluation was completed in validating this measurement scale in a population of cryptoglandular perianal fistula patients. Therefore no comment can be made in this regard.
**Additional comments:**	The EQ-5D provides a standardized and concise measure that allows for comparisons across different health conditions and populations, making it valuable for both clinical research and healthcare decision-making.
**Online example:**	Jayne DG, Scholefield J, Tolan D, Gray R, Senapati A, Hulme CT, Sutton AJ, Handley K, Hewitt CA, Kaur M, Magill L; FIAT Trial Collaborative Group. A Multicenter Randomized Controlled Trial Comparing Safety, Efficacy, and Cost-effectiveness of the Surgisis Anal Fistula Plug Versus Surgeon's Preference for Transsphincteric Fistula-in-Ano: The FIAT Trial. Ann Surg. 2021 Mar 1;273(3):433-441. doi: 10.1097/SLA.0000000000003981. PMID: 32516229.

**Instrument name**	**Wexner Cleveland Clinic score**
**Acronym:**	Wexner
**Domains assessed:**	Incontinence
**Description:**	The Wexner Score consists of a series of questions that evaluate various aspects of bowel control, including the frequency and types of incontinence episodes, the use of pads, and the impact on daily activities.
**Number of items:**	5 items
**Estimated time to complete:**	< 5 minutes
**Administer to:**	Patients
**Require specially trained tester:**	No
**Mode of administration:**	In-person, Online
**License/Purchasing tests/Contact and copyright information/Website:**	This measurement scale has not been licensed.
**Current licensing/Equipment costs:**	This measurement scale has no licensing fees.
**Equipment required/useful:**	In person: Survey form and pen

	Online: Access to a computer or phone with internet access
**Number of published cryptoglandular anal fistula publications using instrument (2008 – 2022):**	Used 69 times in literature for the purpose of scoring Incontinence
**Highest COSMIN rating:**	No evaluation was completed in validating this measurement scale in a population of cryptoglandular perianal fistula patients. Therefore no comment can be made in this regard.
**Additional comments:**	The Wexner score has been widely used to reliably evaluate and quantify the impact of fecal incontinence on patients' lives. This measurement instrument has been validated for the use in patients with fecal incontinence.
**Online example:**	Abramowitz L, Soudan D, Souffran M, Bouchard D, Castinel A, Suduca JM, Staumont G, Devulder F, Pigot F, Ganansia R, Varastet M; Groupe de Recherche en Proctologie de la Société Nationale Française de Colo-Proctologie and the Club de Réflexion des Cabinets et Groupe d'Hépato-Gastroentérologie. The outcome of fistulotomy for anal fistula at 1 year: a prospective multicentre French study. Colorectal Dis. 2016 Mar;18(3):279-85. doi: 10.1111/codi.13121. PMID: 26382623.

**Instrument name**	**Vaizey Incontinence Score**
**Acronym:**	Vaizey Score
**Domains assessed:**	Incontinence
**Description:**	The Vaizey Incontinence Score consists is a questionnaire that includes four key parameters related to fecal incontinence, namely frequency, consistency, control and lifestyle.
**Number of items:**	7 items
**Estimated time to complete:**	< 5 minutes
**Administer to:**	Patients
**Require specially trained tester:**	No
**Mode of administration:**	In-person, Online
**License/Purchasing tests/Contact and copyright information/Website:**	This measurement scale has not been licensed.
**Current licensing/Equipment costs:**	This measurement scale has no licensing fees.
**Equipment required/useful:**	In person: Survey form and pen<br>Online: Access to a computer or phone with internet access
**Number of published cryptoglandular anal fistula publications using instrument (2008 – 2022):**	Used 11 times in literature for the purpose of scoring Incontinence
**Highest COSMIN rating:**	No evaluation was completed in validating this measurement scale in a population of cryptoglandular perianal fistula patients. Therefore no comment can be made in this regard.
**Additional comments:**	The Vaizey score is based on the Wexner score and includes two additional parameters: defecation urgency and the use of constipating medication. It has been widely used to reliably evaluate and quantify the impact of fecal incontinence on patients' lives. This measurement instrument has been validated for the use in patients with fecal incontinence.
**Online example:**	van Praag EM, Stellingwerf ME, van der Bilt JDW, Bemelman WA, Gecse KB, Buskens CJ. Ligation of the Intersphincteric Fistula Tract and Endorectal Advancement Flap for High Perianal Fistulas in Crohn's Disease: A Retrospective Cohort Study. J Crohns Colitis. 2020 Jul 9;14(6):757-763. doi: 10.1093/ecco-jcc/jjz181. PMID: 31696918; PMCID: PMC7346888.

Instrument name	Fecal Incontinence Quality of Life Scale
**Acronym:**	FIQL or Rockwood scale
**Domains assessed:**	Quality of Life

**Description:**	The Fecal Incontinence Quality of Life Scale is composed of a total of 29 items; these items form four scales: Lifestyle (10 items), Coping/Behavior (9 items), Depression/Self-Perception (7 items), and Embarrassment (3 items).
**Number of items:**	29 items
**Estimated time to complete:**	N/A
**Administer to:**	Patients
**Require specially trained tester:**	No
**Mode of administration:**	In-person, Online
**License/Purchasing tests/Contact and copyright information/Website:**	This measurement scale has not been licensed.
**Current licensing/Equipment costs:**	This measurement scale has no licensing fees.
**Equipment required/useful:**	In person: Survey form and pen<br>Online: Access to a computer or phone with internet access
**Number of published cryptoglandular anal fistula publications using instrument (2008 – 2022):**	Used 7 times in literature to evaluate incontinence
**Highest COSMIN rating:**	No evaluation completed in validating this measurement scale in a population of cryptoglandular perianal fistula patients. Therefore no comment can be made in this regard.
**Additional comments:**	This measurement scale was created to evaluate quality of life in patients suffering with fecal incontinence.
**Online example:**	Rockwood TH, Church JM, Fleshman JW, Kane RL, Mavrantonis C, Thorson AG, Wexner SD, Bliss D, Lowry AC. Fecal Incontinence Quality of Life Scale: quality of life instrument for patients with fecal incontinence. Dis Colon Rectum. 2000 Jan;43(1):9-16; discussion 16-7. doi: 10.1007/BF02237236. PMID: 10813117.

**Instrument name**	**Colorectal functional outcome questionnaire**
**Acronym:**	COREFO Questionnaire
**Domains assessed:**	Incontinence
**Description:**	The COREFO questionnaire was specifically designed for patients who have undergone colorectal surgery, encompassing a comprehensive assessment of both incontinence and the functional outcomes experienced by these individuals. This questionnaire offers a multidimensional perspective on patient well-being after intervention.
**Number of items:**	27 items
**Estimated time to complete:**	10-15 minutes
**Administer to:**	Patients
**Require specially trained tester:**	No
**Mode of administration:**	In-person, Online
**License/Purchasing tests/Contact and copyright information/Website:**	This measurement scale has not been licensed.
**Current licensing/Equipment costs:**	This measurement scale has no licensing fees.
**Equipment required/useful:**	In person: Survey form and pen<br>Online: Access to a computer or phone with internet access
**Number of published cryptoglandular anal fistula publications using instrument (2008 – 2022):**	Used 3 times in literature to evaluate incontinence
**Highest COSMIN rating:**	No evaluation completed in validating this measurement scale in a population of cryptoglandular perianal fistula patients. Therefore, no comment can be made in this regard.
**Additional comments:**	This questionnaire has been developed to facilitate the comprehensive evaluation of outcomes across the spectrum of colorectal surgery. While it has proven particularly valuable in assessing the outcomes of colorectal cancer surgeries and the impact of low anterior resection syndrome (LARS), its applicability extends to various other surgical interventions within the colorectal domain.

**Online example:**	van Koperen PJ, Bemelman WA, Bossuyt PM, Gerhards MF, Eijsbouts QA, van Tets WF, Janssen LW, Dijkstra FR, van Dalsen AD, Slors JF. The anal fistula plug versus the mucosal advancement flap for the treatment of anorectal fistula (PLUG trial). BMC Surg. 2008 Jun 23;8:11. doi: 10.1186/1471-2482-8-11. PMID: 18573198; PMCID: PMC2467399.

**Instrument name**	**Anal Fistula Quality of Life Scale**
**Acronym:**	AF-QOL
**Domains assessed:**	Quality of Life
**Description:**	The Anal Fistula Quality of Life Scale is composed of 22 items, consisting of 5 domains:<br>Symptoms (5 items), Daily life and leisure activity (4 items), Work and school/university (2 items), Thoughts regarding surgery an treatment (5 items) (including specific seton domain – 3 items), Impact on relationships (3 items), Impact on relationships (3 items), and Psychological impact (3 items)
**Number of items:**	22 items
**Estimated time to complete:**	5-10 minutes
**Administer to:**	Patients
**Require specially trained tester:**	No
**Mode of administration:**	In-person, Phone, Mail, Online
**License/Purchasing tests/Contact and copyright information/Website:**	This measurement scale has not been licensed.
**Current licensing/Equipment costs:**	This measurement scale has no licensing fees.
**Equipment required/useful:**	In person: Survey form and pen<br>Online: Access to a computer or phone with internet access
**Number of published cryptoglandular anal fistula publications using instrument (2008 – 2022):**	N/A<br>Not published in this period
**Highest COSMIN rating:**	N/A
**Additional comments:**	This measurement instrument was created to evaluate quality of life in patients with cryptoglandular anal fistulas. It was created with input from patients with and has been validated in a sample of patients with cryptoglandular anal fistula. The final score is on a scale of 0 – 100, where 0 implies poor and 100 the best possible quality of life.
**Online example:**	N/A
