# Supplementary material for: Cryptoglandular Anal Fistula Core Outcome Measurement Set (AFCOMS): standardised definitions and measurement instruments
Source: eClinicalMedicine. 2026 Feb 6;92:103745. doi: 10.1016/j.eclinm.2025.103745 (PMC12907631; doi:10.1016/j.eclinm.2025.103745)
Supplement: Collab authors [file mmc2.docx]

**AFCOMS Consensus Meeting Collaborators**

| **First names** | **Surnames** |
| --- | --- |
| Ademola | Adeyeye |
| Andrea Marco | Tamburini |
| Angelo Alessandro | Marra |
| Anthony | Lin |
| Arda | Isik |
| Eleni | Andriopoulou |
| Fatima | Senra |
| Flavia | Alexandre |
| Francesco | Pata |
| Gaetano | Gallo |
| Gabriele | Bislenghi |
| Jasper | Stijns |
| Jennie | Grainger |
| Jesus | Lopez-Alcalde |
| Johannes | Jongen |
| Lilli | Lundby |
| Lillian | Reza |
| Martijn | Gosselink |
| Peter | Ambe |
| Philip | Lung |
| Raimund | Strouhal |
| Ricardo | Rocha |
| Giulio Aniello | Santoro |
| Stephen | Ward |
